# A systems biology approach to define SARS-CoV-2 correlates of protection

**DOI:** 10.1038/s41541-025-01103-2

**Published:** 2025-04-14

**Authors:** Caolann Brady, Tom Tipton, Oliver Carnell, Stephanie Longet, Karen Gooch, Yper Hall, Javier Salguero, Adriana Tomic, Miles Carroll

**Affiliations:** 1 Centre for Human Genetics, Nuffield Department of Medicine, University of Oxford, Oxford, United Kingdom; 2 Pandemic Sciences Institute, University of Oxford, Oxford, United Kingdom; 3 UK Health Security Agency; Porton Down, Salisbury, United Kingdom; 4 International Center for Infectiology Research (CIRI), Team GIMAP, Claude Bernard Lyon 1 University, Saint-Etienne, France; 5 National Emerging Infectious Diseases Laboratories, Boston, MA USA; 6 Department of Virology, Immunology & Microbiology, Boston University Medical School, Boston, MA USA; 7 Biomedical Engineering, Boston University, College of Engineering, Boston, MA USA

**Keywords:** Immunology, Microbiology

## Abstract

Correlates of protection (CoPs) for SARS-CoV-2 have yet to be sufficiently defined. This study uses the machine learning platform, SIMON, to accurately predict the immunological parameters that reduced clinical pathology or viral load following SARS-CoV-2 challenge in a cohort of 90 non-human primates. We found that anti-SARS-CoV-2 spike antibody and neutralising antibody titres were the best predictors of clinical protection and low viral load in the lung. Since antibodies to SARS-CoV-2 spike showed the greatest association with clinical protection and reduced viral load, we next used SIMON to investigate the immunological features that predict high antibody titres. It was found that a pre-immunisation response to seasonal beta-HCoVs and a high frequency of peripheral intermediate and non-classical monocytes predicted low SARS-CoV-2 spike IgG titres. In contrast, an elevated T cell response as measured by IFNγ ELISpot predicted high IgG titres. Additional predictors of clinical protection and low SARS-CoV-2 burden included a high abundance of peripheral T cells. In contrast, increased numbers of intermediate monocytes predicted clinical pathology and high viral burden in the throat. We also conclude that an immunisation strategy that minimises pathology post-challenge did not necessarily mediate viral control. This would be an important finding to take forward into the development of future vaccines aimed at limiting the transmission of SARS-CoV-2. These results contribute to SARS-CoV-2 CoP definition and shed light on the factors influencing the success of SARS-CoV-2 vaccination.

## Introduction

Severe acute respiratory coronavirus 2 (SARS-CoV-2) infection, the aetiological agent of coronavirus disease 2019 (COVID-19), was de-escalated by the World Health Organisation from ‘Public Health Emergency of International Concern’ (PHEIC) status in May 2023. This recommendation by the WHO is largely because of the successful development and deployment of effective SARS-CoV-2 vaccines. Despite real-world vaccine efficacy data, clinical trial data and data on passive immunisation demonstrating that spike antibody titres are associated with protection from SARS-CoV-2^1–17^, a correlate of protection (CoP), ‘the immune response that is responsible for and interrelated with protection^18^,’ for SARS-CoV-2 has not yet been defined. This complicates our understanding of SARS-CoV-2 immunity and could slow the approval of future vaccines to SARS-CoV-2 variants of concern (VOCs).

Knowledge of the influenza CoP has been central to seasonal influenza vaccine development^19^. Equally, serum bactericidal antibody (SBA) ≥ 1:4, identified as the CoP for meningococcal serogroup C in military recruits in 1969^20^, remains the gold standard for meningococcal vaccine evaluation and approval. The insight provided by animal models for the definition of a CoP, is evident from research into the *Bordetella pertussis* CoP. Firstly, a non-lethal aerosol model for *B. pertussis* colonisation in mice identified that convalescent serum transfer did not impact bacterial control, whereas CD4^+^ T cell adoptive transfer engendered bacterial clearance and CD4^+^ T cell depletion abrogated protection^21,22^. A later non-human primate model confirmed that a Th17/Th1 response was crucial for the clearance of infection and prevention of transmission, providing rationale behind the suboptimal efficacy of the acellular vaccine that induced a Th2/Th1-biased response^23^.

Several barriers exist for defining a SARS-CoV-2 CoP in humans. SARS-CoV-2 vaccine efficacy was determined based on the reduced rates of infection in the vaccinated versus placebo control groups, however these trials did not include a challenge component^24–28^. It is therefore plausible that, because of government-imposed lockdowns and social distancing measures, most of the trial participants were not exposed to SARS-CoV-2. This uncertainty surrounding vaccinee or convalescent exposure complicates the definition of ‘protection’ versus ‘failed protection’ outcomes, hence the immune response associated with protection cannot accurately be deduced.

Asymptomatic disease further complicates the identification of protected versus unprotected endpoints. In many phase II clinical trials PCR testing was encouraged for those presenting with COVID-19 symptoms, with less frequent routine testing in place to capture potential asymptomatic cases. In the case of ‘sterilising immunity,’ serological evidence of infection (ie. a SARS-CoV-2 nucleocapsid IgG response) may be lacking, further limiting our ability to identify instances of ‘protection.’

Many of the issues described above can be overcome by SARS-CoV-2 human challenge studies^29–31^. However, sustained infection could not be established in participants with pre-existing immunity to SARS-CoV-2 despite dose escalation^31^, and SARS-CoV-2 naïve participant infection rates were low (53%)^29^. Therefore, the sample size of SARS-CoV-2 infected individuals is low, reducing the power of the study. Human safety considerations and cost are additional challenges associated with performing controlled human infection studies. Animal models overcome many of these limitations. It is also possible to study the relevant tissue in animal challenge studies, which provides additional insights into the pathological burden following challenge. Additional advantages of animal models include their better-documented immunological history, the ability to track immune and viral trajectories following vaccination or challenge, and the scope to test suboptimal vaccines. The advantages of pre-clinical models for the definition of CoPs are more extensively reviewed in Brady et al.^32^.

Multiparameter immunological datasets, like the dataset we have here, are the foundation of systems immunology. Systems immunology considers the challenge model immune response as a whole dynamic system, rather than focusing on single static immune parameters^33^. Knowledge extraction from high dimensional datasets can be assisted by machine learning (ML), which identifies patterns in the data to draw links between input features and outcomes. In the context of vaccine research, ML can be applied to identify the immune response post-immunisation that predicts protection, the primary requirement of a disease CoP. ML has been applied in many systems immunology studies to identify the immune responses that predict vaccine efficacy including for influenza and yellow fever vaccines^34,35^ and has been extensively reviewed by Davis and Pulendran et al.^33^.

The analysis reported here is the largest compilation of pre-clinical SARS-CoV-2 vaccine trial and re-challenge study data described to date (n = 90). All pre-clinical research involved rhesus (RhM) or cynomolgus macaques (CyM) (shown to be comparable COVID-19 models by Salguero et al.^36^) and was performed at the same facility (UKHSA, Porton Down, Salisbury). These studies investigated both optimal and sub-optimal dosing of SARS-CoV-2 vaccine candidates and, importantly, used the same challenge regimen, including matched viral inocula and viral challenge routes, making challenge outcome data comparable. Using a ML platform named Sequential Iterative Modelling Overnight (SIMON), to identify the algorithms that best model the post-challenge outcomes, we report the immune parameters that predict clinical protection, upper respiratory tract (URT) and lower respiratory tract (LRT) viral control, and the early immune predictors of high spike IgG titres post-immunisation.

## Results

### Immunological screen of non-human primates post-vaccination and primary challenge show heterogenous immune responses and outcomes to SARS-CoV-2

**Fig. 1:**
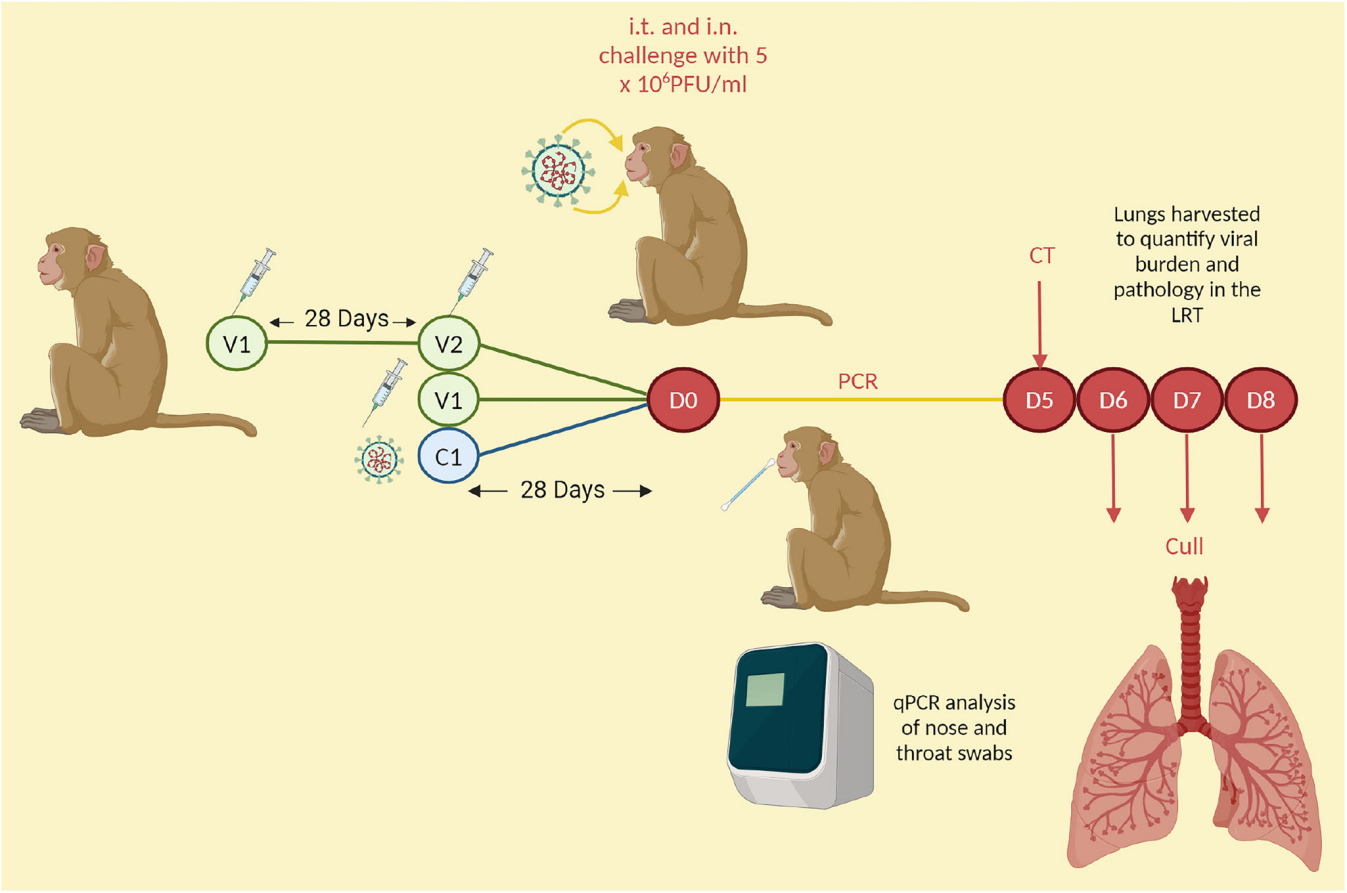
Schematic of pre-clinical study timeline. The day of challenge post-immunisation is D0. The immunisation strategies varied. For two dose regimens, there was a 28 day interval between vaccine dose one (V1) and two (V2). There was a 28 day interval between second/only vaccine dose and challenge, in all but the FIV group, that were challenged 14 days post-FIV vaccination. There was also a 28 day interval between the primary challenge (C1) and secondary challenge (D0) in the re-challenged cohort. During acute infection, nasal and throat swabs were collected for qPCR. Animals were culled 6-8 days (D6-D8) post-challenge, and the lungs were harvested.

A cohort of ninety RhMs and CyMs either received a primary challenge, one or two doses of a vaccine candidate (an mRNA vaccine, a DNA vaccine, a formalin-inactivated vaccine (FIV) or the ChAdOx-vectored vaccine^36–40^, Table 1) or were in the control arm of the studies and were not immunised. Animals were subsequently challenged or re-challenged with a high viral dose of SARS-CoV-2 (5 ×10^6^ PFU of Victoria/1/2020), delivered intratracheally and intranasally. There was a 28-day interval between the second/only vaccine dose and challenge in all but the FIV group that were challenged 14 days post-FIV vaccination. There was also a 28-day interval between the primary challenge and secondary challenge in the re-challenge cohort (Fig. 1). During acute infection, nasal and throat swabs were collected for qPCR. The lungs of each animal were harvested six-to-eight days post-challenge, for histopathology assessment, and qPCR of viral genomic RNA (gRNA) was performed on bronchoalveolar lavage (BAL) fluid to ascertain viral burden in the lung. The ChAdOx study and RhM versus CyM study included animals that were culled at earlier or later timepoints and these animals were excluded from the analysis of clinical and lung viral load outcomes (see Table 2 for group breakdown). Lung histopathology was scored using a scoring system defined by Salguero et al.^36^, which involved the scoring of seven lung pathology parameters from zero to four, with a score of four corresponding to more marked pathology, and a cumulative score of 28, across all parameters, describing maximum severity^36^.

**Table 1:** Summary of vaccines included in study

Manufacturer	Platform	Adjuvant	Antigen	Delivery	Citation
CureVac	Non-nucleoside modified mRNA	LNP	Pre-fusion stabilised full-length spike	Intramuscular	^22^
Inovio	DNA	Unknown	Full-length WT spike	Intradermal with CELLECTRA-ID electroporation technology	^21^
Oxford-AstraZeneca	ChAd vector	Adenoviral vector	Pre-fusion stabilised full-length spike	Intramuscular	^24^
Oxford-UKHSA	Formalin-inactivated virus	Alhydrogel	Whole SARS-CoV-2	Intramuscular	^23^

Manufacturer, vaccine platform, adjuvant, antigen and delivery mechanisms included.

**Table 2:** Number of macaques in each subgroup of study for each post-challenge outcome investigated

	Unvaccinated	Low Dose mRNA	High Dose mRNA	One Dose DNA	Two Dose DNA	FIV	Re-Challenge	ChAd-Vectored
Clinical Outcome	35	6	6	6	6	6	6	6
LRT Viral Load	31	6	6	6	6	6	6	2
URT Viral Load	48	6	6	6	6	6	6	6

Serology data, including spike and receptor binding domain (RBD) binding antibody measured by a standard ELISA protocol, standardised seasonal human coronavirus (HCoV) antigen binding antibody measured by MSD, standardised Ig isotype and IgG subclass titres measured by a multiplex bead assay and neutralising antibody measured by micro neutralisation assay (MNA), was collected across all preclinical studies and the data is summarised in Supplementary Figs. 1, 2, 3 and 4, respectively. SARS-CoV-2-reactive T cell data measured by a standard IFNγ ELISpot protocol, and whole blood immunophenotyping data was also collected for each of the preclinical studies, except the re-challenge study. The ELISA, MNA, ELISpot and whole blood immunophenotyping was described and reported in the original pre-clinical publications^36–40^. In our study, data from these pre-clinical publications has been compiled along with pre-immunisation serology, spike antibody isotype and IgG subclass data to investigate SARS-CoV-2 CoPs.

**Fig. 2:**
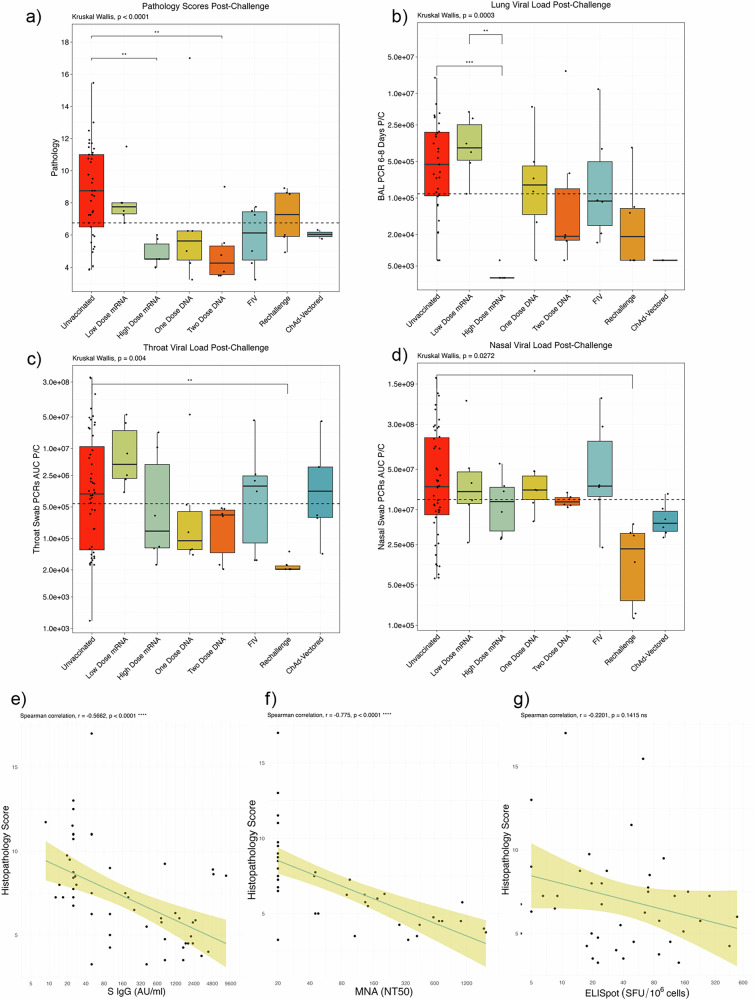
SARS-CoV-2 challenge outcome analysis. Box and whisker plots demonstrating the success of each immunisation strategy for **a** clinical protection determined using a histopathology scoring system, **b** limiting lung viral burden determined by BAL PCR 6-8 days post-challenge, **c** controlling throat viral load over the course of infection (area-under-the curve of the throat swab PCRs) (**d**) controlling nasal viral load over the course of infection (area-under-the curve of the nasal swab PCRs). Each datapoint represents an animal. Box plots show the group median and inter-quartile range (IQR), and whiskers connect the maximum and minimum values, extending no further than 1.5x IQR (data beyond whiskers are outliers). Kruskal Wallis test and Dunn’s multiple comparisons test performed. Two-tailed Spearman correlations between lung histopathology scores and immune responses with 95% confidence intervals (**e**) spike IgG, **f** neutralising antibody, **g** ELISpot. AUC area under the curve, P/C post-challenge.

The range of vaccine platforms, doses and regimens and the inclusion of a re-challenge cohort serves as an opportunity to compare pathology outcomes following SARS-CoV-2 challenge and identify shared CoPs (Fig. 2). Here, we focus on 4 possible outcomes to describe the protection against severe infection; LRT disease burden, evaluated by the 1) lung histopathology scores and 2) viral load in the lungs, and protection against URT infection measured by 3) nasal and 4) throat viral loads.

It was found that two high doses of the mRNA vaccine and two doses of the DNA vaccine protected against severe infection as measured by the histopathology scores (Dunn’s multiple comparisons test, p = 0.0025 and p = 0.0074, Fig. 2a).

BAL qPCR 6-8 days post-challenge of macaques that received two high doses of the mRNA vaccine significantly differed to the unvaccinated control group (Dunn’s multiple comparisons test, p = 0.0025, Fig. 2b).

Interestingly, only immunity generated after natural infection was able to protect against URT infection as indicated by the nose and throat PCR area under the curve (AUC) data (Figs. 2c and 2d). Only re-challenged macaques had significantly lower viral AUCs to the unvaccinated control group (Dunn’s multiple comparisons test, *p* = 0.0246 and *p* = 0.0064, respectively, Figs. 2c and 2d). Re-challenge macaques also had significantly lower throat viral load AUCs than the macaques that received two low dose mRNA vaccines (Dunn’s multiple comparisons test, *p* = 0.0019, Figs. 2c and 2d).

SARS-CoV-2 specific humoral and T cell responses have been associated with the outcome of vaccine pre-clinical studies, clinical trials and vaccine follow-up studies^2,15,40–50^. To identify shared immunological signatures associated with protection, we performed integrated analysis across vaccine platforms. We first performed correlation analysis using immunogenicity data collated from the vaccine and challenge trials discussed above. We observed a significant positive correlation between histopathology scores and spike IgG (Fig. 2e; Spearman correlation, r = −0.5662, *p* < 0.0001), receptor-binding domain (RBD)-binding IgG (Supplementary Fig. 5; Spearman correlation r = −0.5059, *p* = 0.0001) and NAbs (Fig. 2f; Spearman correlation, r = −0.775, *p* < 0.0001), but an insignificant relationship between histopathology scores and IFNγ ELISpot (Fig. 2g; r = −0.2201, *p* = 0.1415).

Vaccine outcome parameters of interest included histopathology score, lung viral load 6-8 days post-infection, throat viral load AUC during infection and nasal viral load AUC during infection. To support ML predictions, each of these outcomes were made binary; either protected or pathology, and either high or low virus. Each macaque was assigned to a binary category based on defined cut-offs (histopathology score = 6.75, lung viral load AUC = 120,000, throat viral load AUC = 590,000 and nasal viral load AUC = 1.5 ×10^7^), for each outcome parameter (Supplementary Fig. 6–9). Critically, depending on the outcome parameter used to define vaccine efficacy in pre-clinical models, the immunisation strategies varied in success. An immunisation strategy that minimised pathology post-challenge did not necessarily mediate viral control. Only 19 out of a total of 69 macaques (27.5%), which had data on each of the four post-challenge outcomes, had agreeable virology outcomes and clinical outcomes. Clinical outcomes were the most agreeable with the BAL PCR 6-8 days post-challenge outcome, with 49 of 69 macaques having high lung virus plus high pathology or low lung virus plus low pathology (71%). There was also a strong significant positive correlation between lung viral load and histopathology scores (Supplementary Fig. 10a; Spearman correlation, r = 0.4782, *p* < 0.001). However, histopathology scores did not correlate strongly with throat viral load (Supplementary Fig. 10b, Spearman correlation, r = 0.1233, *p* = 0.3096) and correlated weakly with nasal viral load (Supplementary Fig. 10c, Spearman correlation, r = 0.2394, *p* = 0.0444). Therefore, clinical protection (ie. protection from pathology) and protection from high viral load, particularly in the upper respiratory tract, may need to be considered separately.

### Immunological signatures that differentiate mild from more severe pathology outcomes post-SARS-CoV-2 challenge

Macaques do not experience severe COVID-19 symptoms or succumb to infection, therefore in this study, clinical outcomes were stratified by histopathology scores that were assigned to macaques following histopathological assessment of respiratory organs 6-8 days post-SARS-CoV-2 challenge. The histopathology scoring system is described in Salguero et al.^36^. Based on these scores the macaques were classified into protected or pathology groups (cut-off = 6.75, Supplementary Fig. 6).

In-life computed tomography (CT) imaging scores day 5 post-challenge was used to define clinical severity in animals that were not assigned a histopathology score (n = 6, from the control group in the re-challenge study), or were culled 13-14 days post-challenge (n = 4 from ChAd-vectored vaccinated group and n = 4 from the ChAd-vectored control group), because at this timepoint, the infection would have resolved and the histopathology score would not accurately reflect acute pathology. Animals that were not culled between the timeframe of 6-8 days post-SARS-CoV-2 challenge, and did not have a CT scan performed during acute infection, were excluded from this analysis (n = 12, see Table 2 for group breakdown). CT scores correlated with histopathology score (Pearson correlation, r = 0.624 and *p* < 0.0001) and there was strong agreement between protection or pathology group assignment when histopathology or CT score were used (71%, and of the 29% with conflicting categorisation, 50% of macaques had scores around the cut-off ( ± 1.5)). The ChAdOx study and re-challenge control macaques, for which we relied on CT scores alone for clinical outcome classification, had CT scores that were far from the median CT score cut-off, therefore we were confident in the placement of these macaques into protected and pathology groups based on their CT score. Correlation filtering (corr) was not performed as a) immune features commonly correlate and b) all immunological measurements pre-challenge were included in the dataset, and so inter-timepoint correlations would exist (see Supplementary Fig. 11).

**Fig. 3:**
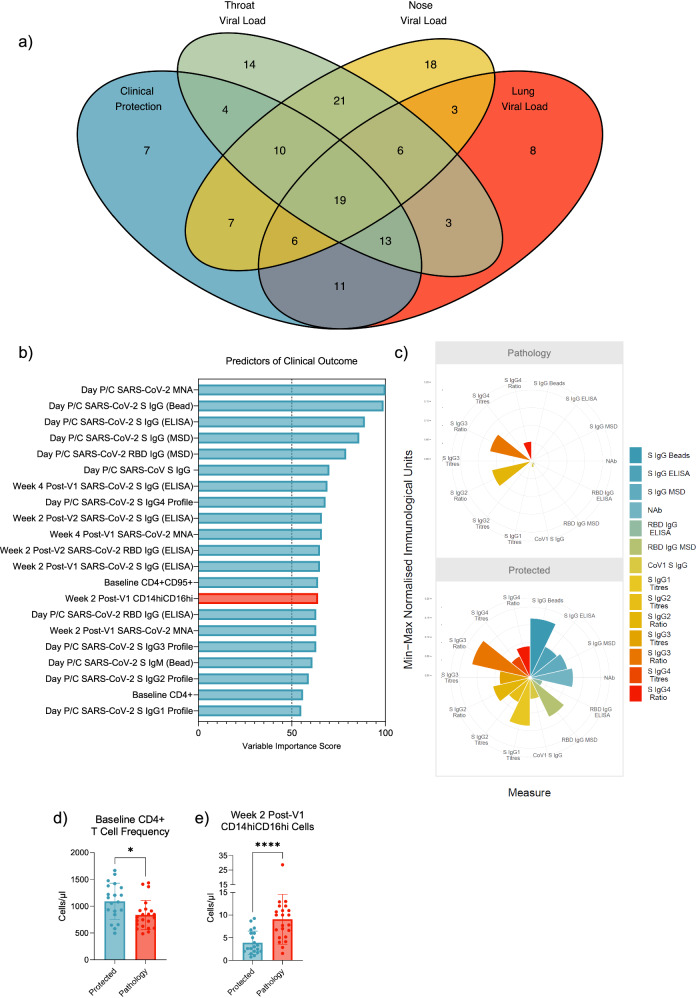
SARS-CoV-2 challenge clinical outcome analysis. **a** Venn diagram showing the agreeance, or lack thereof, between each outcome post-challenge. **b** Variables of importance for clinical protection – predictors of protection in blue and predictors of pathology in red. **c** Polar plot showing the difference in magnitude of the humoral responses between the protected and pathology groups. The abundance of (**d**) baseline CD4^+^ T cells phenotyped by flow cytometry predicting protection and **e** CD14^hi^CD16^hi^ monocytes two weeks post-V2 predicting pathology. Datapoints represent each animal, and the bars show group means with standard deviation. Two-sided Mann Whitney test performed.

Using a supervised ML platform, SIMON, 171 machine learning algorithms were tested and 26 successful models were identified based on model selection criteria of having an area under receiver operator curve (AUROC) for the training set that is greater than 0.7 and a test set AUROC greater than the training AUROC. The best performing model was built using the Boosted Classification Trees (ada) algorithm (train AUROC 0.8771 and test AUROC 0.9074, Supplementary Fig. 12). Of the 158 immune features provided, 21 immune features scored a variable of importance score above 50, and so best predict clinical protection when modelled with the ada algorithm (Fig. 3b, Supplementary Fig. 13).

SARS-CoV-2 neutralising antibody (NAb) titres best modelled protection from SARS-CoV-2 challenge, as the highest variable importance score (VIS) was recorded for this feature (VIS 100, Figs. 3b and 3c) and high NAb titres differentiated ‘protected’ from ‘pathology’ outcomes with significance (p < 0.0001, Supplementary Fig. 13). High SARS-CoV-2 spike binding IgG titres were also strong predictors of protection. High SARS-CoV-2 spike IgG binding titres on the day of challenge measured by three different serological assays were variables of importance (VOI) and significantly differed between the ‘protected’ and ‘pathology’ groups; spike-conjugated bead assay (VIS 99, *p* < 0.0001), ELISA (VIS 89, *p* < 0.0001), and MSD (VIS 86, *p* < 0.0001, Supplementary Fig. 13), validating its prediction potential, even when measured by different assays. A high RBD IgG response on the day of challenge was also predictive of clinical protection (MSD; VIS 79, *p* < 0.0001, ELISA; VIS 63, *p* = 0.0002, Supplementary Fig. 13). High SARS-CoV-1 spike IgG (VIS 70, *p* < 0.0001, Supplementary Fig. 13) also predicted outcome upon challenge.

As early as two weeks post-vaccine dose one (V1), high spike IgG and NAb titres predicted protection (spike IgG by ELISA VIS 65, and NAbs by MNA VIS 63) and there was a significant difference in spike IgG (*p* = 0.0184) and NAb (*p* = 0.0032) titres between the pathology and protected groups from this timepoint (Supplementary Fig. 13). High spike-binding and neutralising antibody titres from week four post-V1 (S IgG; VIS 69, p = 0.003, MNA; VIS 66, *p* = 0.0004, Supplementary Fig. 13) also contributed to the modelling of protection and significantly differentiated protected from pathology groups. Similarly, from week two post-V2, high spike IgG titres and RBD IgG titres predicted clinical protection (spike IgG ELISA; VIS 66, *p* < 0.0001, RBD IgG ELISA; VIS 65, *p* < 0.0001, Supplementary Fig. 13). Early serological predictors of clinical protection were investigated further to ensure this result was not influenced by outliers, which was not the case (see matched timecourse graphs, Supplementary Figs. 14a–c).

IgG subclasses also emerged as predictors of protection by SIMON analysis. IgG subclass titre and its ratio with respect to the other subclasses, was incorporated into the one parameter for SIMON analysis (Supplementary Fig. 15). Protected macaques fell into IgG4 and IgG3 subclass categories with higher titres and higher ratios (Fig. 3c), which significantly differed from the IgG subclass category distribution of macaques in the pathology group (Chi-square test); IgG4 (VIS 68, *p* = 0.0029, Supplementary Fig. 13) and IgG3 (VIS 63, *p* = 0.0026, Supplementary Fig. 13). Protected macaques fell into IgG2 and IgG1 high titre categories (Fig. 3c), which significantly differed from the IgG subclass distribution of macaques in the pathology group (Chi-square test); IgG2 (VIS 59, *p* = 0.0011, Supplementary Fig. 13) and IgG1 (VIS 55, *p* = 0.0025, Supplementary Fig. 13). High IgM titre was also a predictor (VIS 61) and titres significantly differed between the protected and pathology groups (*p* = 0.0001, Supplementary Fig. 13).

The ada algorithm also modelled protection using cellular responses. An early T cell response was important in establishing a protection-associated immune profile as a high abundance of CD4^+^ (VIS 56, Fig. 3d) and CD4^+^CD95^+^ (VIS 64, Supplementary Fig. 13) T cells at baseline were predictors of protection and significantly differed between protected and pathology groups (*p* = 0.015 and *p* = 0.0265, respectively). A high frequency of CD14^hi^CD16^hi^ cells at two weeks post-V2, was the only parameter predictive of pathology (VIS 64) and significantly differentiated pathology from protected groups (*p* < 0.0001) (Fig. 3e).

No singular immunisation regimen contributed to the trend of immune divergence that determined predictability (Supplementary Fig. 14d–f), therefore the predictors identified are not vaccine-specific. Clinical outcome could not be modelled using MNA titres alone, or spike IgG titres alone, with the ada algorithm. However, MNA and spike IgG titres together successfully modelled clinical protection with improved model performance (train AUROC of 0.9146 and test AUROC of 0.9444, data not shown). This highlights the potential for enhanced predictive power when a number of predictive immune parameters are used together to predict post-challenge outcomes.

### Immunological signatures that differentiate low from high viral loads in the lung post-cull following SARS-CoV-2 infection

A major benefit of using animal models for the study of SARS-CoV-2 CoPs is that we can monitor the extent of viral control following a known exposure event with a known titre of virus.

qPCR on BAL fluid to measure SARS-CoV-2 viral RNA burden in the lung has been used by several groups as a protective endpoint in pre-clinical evaluation of candidate SARS-CoV-2 vaccines^42,51^. The frequency of macaques having both high lung viral load and pathology is 71% (Fig. 3a) and there was a significant correlation (r = 0.4782, *p* < 0.0001, Spearman Correlation, Supplementary Fig. 10a) between these outcomes. The relationship between virology and pathology in the lung, may therefore mean that LRT viral load burden can be used as a surrogate for severity of pathology. Therefore, *in lieu* of a veterinary pathologist or a robust histopathology scoring system, BAL PCR results can be reflective of clinical protection as was applied in mRNA-1273 pre-clinical trials^42,51^. We next look to determine if the immune parameters that predicted pathology also predicted viral control in the lung.

**Fig. 4:**
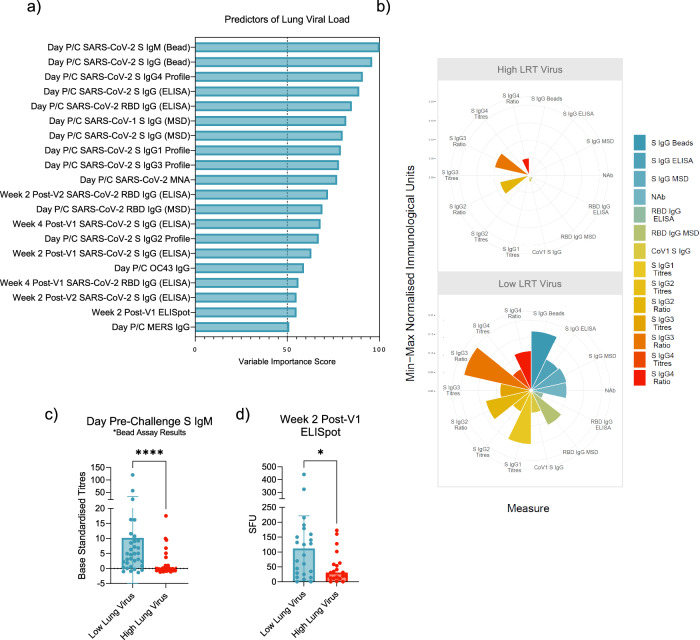
Immune predictors of lung viral load post-SARS-CoV-2 challenge. **a** Variables of importance for lung viral load –predictors of low viral load in blue. **b** Polar plot showing the difference in magnitude of the humoral responses between the low and high lung viral load groups. **c** Spike IgM on the day pre-challenge predicts low lung virus (**d**) SARS-CoV-2 reactive PBMCs two weeks post-V1 predict low lung virus. Datapoints in bar charts represent each animal, and bars show group means with standard deviation. Two-sided Mann Whitney test performed.

Of the 90 macaques, 69 had BAL qPCR data from 6-8 days post-challenge available (see Table 2 for group breakdown). Immune features, measured at all timepoints pre-challenge, were used in the attempt to model lung viral burden. SIMON used 174 machine learning algorithms and found 10 algorithms that successfully modelled viral titres in the lung. The best performing model was built using the LogitBoost algorithm (train AUROC 0.7847 and test AUROC 0.8214, Supplementary Fig. 12) and 20 of the 158 immune features were identified as predictors of lung viral load outcomes (Fig. 4a).

Many of the same parameters that predicted clinical protection, also predicted low lung virus (Fig. 4b). High SARS-CoV-2 spike IgM on the day of challenge was identified as the best predictor of low lung virus and titres significantly differed between macaques with high and low lung viral load (VIS 100, *p* < 0.0001, Fig. 4c, Supplementary Fig. 16). High spike IgG on the day of challenge was also predictive of low lung viral load and titres significantly differed between the low and high lung viral load groups (Bead; VIS 96, *p* < 0.0001, ELISA; VIS 89, *p* < 0.0001 and MSD; VIS 80, *p* = 0.0004, Fig. 4b, Supplementary Fig. 16). High RBD IgG titres, measured by ELISA and MSD on the day of challenge predicted lung viral burden (ELISA; VIS 85, *p* < 0.0001 and MSD; VIS 69, *p* = 0.0019, Fig. 4b, Supplementary Fig. 16), as did high NAb titres (VIS 77, *p* < 0.0001, Fig. 4b, Supplementary Fig. 16). Similar to the predictors of clinical protection, high spike IgG responses as early as two weeks post-V1 could predict low viral load in the lung post-challenge (VIS 63, *p* = 0.0235), as could high spike IgG titres four weeks post-V1 (VIS 68, *p* = 0.0057) and two weeks post-V2 (VIS 55, *p* = 0.0039). High RBD IgG titres from four weeks post-V1 (VIS 56, *p* = 0.0043) and two weeks post-V2 (VIS 72, *p* = 0.0001) were also identified as early predictors of low lung viral load post-challenge (Supplementary Fig. 16). The identification of early serology markers as lung viral load predictors was investigated further to ensure this result was not influenced by outliers, which was not the case (see timecourse graphs, Supplementary Figs. 17a and b). No singular immunisation regimen contributed to the trend of immune divergence that determined predictability (Supplementary Fig. 17c–e). Lung viral load outcome could not be modelled using spike IgM titres alone, spike IgG titres alone or MNA titres alone with the LogitBoost algorithm.

The protective humoral response on the day of challenge was also reactive to other beta CoV spike proteins; SARS-CoV-1 (VIS 82, p = 0.0006, Fig. 4b, Supplementary Fig. 16), HCoV-OC43 (VIS 59, p = 0.0036, Supplementary Fig. 16) and MERS-CoV (VIS 51, p = 0.0513, Supplementary Fig. 16). As per the predictors of clinical protection, IgG subclass also emerged as a predictor of low lung viral load. Higher titres and ratios of IgG4 (VIS 91, p = 0.0023), IgG3 (VIS 78, p = 0.0028) and IgG2 (VIS 67, p = 0.0129) were associated with low lung viral load (Chi-square test, Fig. 4b, Supplementary Fig. 16). High IgG1 titres were also associated with low lung viral load (VIS 79, p = 0.0036, Chi-square test, Fig. 4b, Supplementary Fig. 16).

We did not find CD14^hi^CD16^hi^ monocytes or early T cell subset frequencies to be predictive of lung viral load. Instead, we observed SARS-CoV-2 reactive PBMCs, measured by IFNγ ELISpot two weeks post-V1, to positively predict low lung virus and significantly differentiate macaques that went on to have high or low lung viral burden (VIS 55, p = 0.0329, Fig. 4d).

### Immunological signatures that differentiate low from high viral loads in the upper respiratory tract over the course of acute SARS-CoV-2 infection

Viral control in the upper respiratory tract and the immune factors facilitating this, may also be critical in understanding the endpoint of reduced SARS-CoV-2 transmission. Limiting viral transmission, as a vaccination endpoint secondary to clinical protection from severe disease, was less of a focus in the midst of the pandemic, as it was difficult to ascertain at the clinical trial phase because of the SARS-CoV-2 control measures in place at the time. Whilst the macaque studies reported here did not measure transmission directly in a horizontal transmission setting, lower viral titres in the nose and throat in the 6-8 days post-challenge would be indicative of reduced transmission risk, based on the increased likelihood of transmission via air droplets from sneezing and coughing were viral load at these mucosal sites high.

It is also important to note the poor correlation between histopathology scores and throat viral titres (r = 0.123, p = 0.3096, Spearman Correlation, Supplementary Fig. 10b), and histopathology scores and the nasal viral titres (r = 0.2394, p = 0.0444, Spearman Correlation, Supplementary Fig. 10c), signifying the lack of agreement between these endpoints. The overlap between the virology and clinical outcomes, i.e. the overlap between the macaques in low URT virus and protected groups, or high URT virus and pathology groups, was also unconvincing, 59.7% for throat virus and 54.5% for nose virus (Fig. 3a). This supports the treatment of clinical and URT viral load as distinct outcomes.

**Fig. 5:**
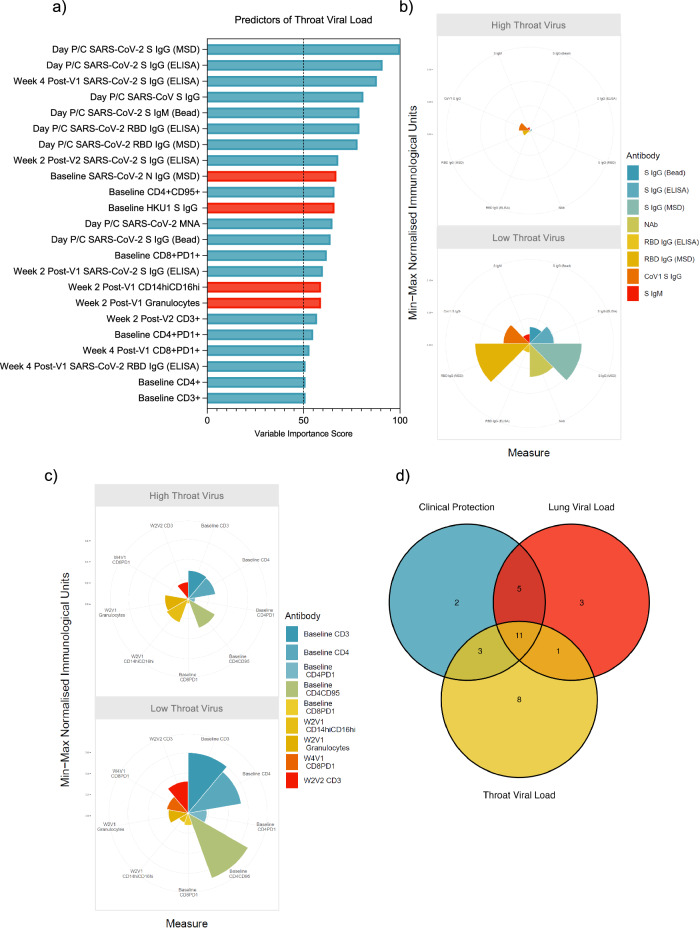
Protection against high viral burden in the upper respiratory tract. **a** Variables of importance for throat viral control – predictors of low viral load in blue and predictors of high viral load in red. **b** Polar plot showing the difference in magnitude of the humoral responses between the low and high viral load groups. **c** Polar plot showing the difference in magnitude of the cellular frequency between the low and high viral load groups. **d** Venn diagram showing the overlap of variables of importance for each of the modelled outcomes.

Models were successfully built for throat virus control only. All 90 macaques had throat swabs collected over the course of infection and AUC was calculated using the available throat swab PCR data. Of 174 machine learning algorithms, 6 models of viral titres in the throat were successfully built. The best performing model was built using the Boosted Classification Trees (ada) algorithm (train AUROC 0.7684 and test AUROC 0.7901, Supplementary Fig. 12) which identified 23 immune features with a VIS greater than 50 (Fig. 5a).

Humoral responses on the day of challenge predicted low throat viral load over the course of acute SARS-CoV-2 infection in macaques (Fig. 5b). High spike IgG on the day of challenge, measured by three different assays, were predictors of low throat viral loads (MSD; VIS 100, p < 0.0001, ELISA; VIS 91, p = 0.0002, Bead Assay; VIS 64, p = 0.0053, Fig. 5b and Supplementary Fig. 18) as were high RBD-binding IgG titres on the day of challenge (ELISA; VIS 79, p = 0.0002, MSD; VIS 78, p = 0.0004, Fig. 5b and Supplementary Fig. 18). High NAb titres on the day of challenge also predicted throat viral control and significantly differentiated low from high viral loads (VIS 65, p = 0.0017, Fig. 5b and Supplementary Fig. 18). The humoral response that supports throat viral control was also reactive to SARS-CoV-1 spike protein (Fig. 5b and Supplementary Fig. 18; VIS 81, p = 0.0003). High spike-binding IgM on the day of challenge also predicted low throat viral loads post-challenge (Fig. 5b and Supplementary Fig. 18; VIS 79, p = 0.0023).

From two weeks post-V1, a high spike-binding antibody titre was predictive of low throat viral load (ELISA; VIS 60, p = 0.0116, Supplementary Fig. 18). Antibody titres at later timepoints post-vaccination had higher variable importance scores and so are of greater importance in the modelling of throat viral load outcomes. For example, spike-binding IgG, measured by ELISA, four weeks post-V1 had a VIS of 88 and two weeks post-V2 had a VIS of 68, and both significantly differentiated low from high throat viral load groups (p < 0.0001 and p = 0.0005, respectively, Supplementary Fig. 18). RBD-binding IgG four weeks post-V1 had a VIS of 51 and significantly differentiated low from high throat viral load groups (p = 0.0004, Supplementary Fig. 18). To ensure these serological parameters were not identified as predictors of throat viral load because of outliers, time-course plots were generated (see Supplementary Fig. 19a and b). No singular immunisation regimen contributed to the trend of immune divergence that determined predictability (Supplementary Fig. 19c–e). Throat viral load outcome could not be modelled using spike IgG titres alone or MNA titres alone with the ada algorithm.

Higher pre-immunisation (study baseline) IgG responses to HKU1 spike and SARS-CoV-2 nucleocapsid were also predictive of high viral load in the throat over the course of infection; HKU1 spike IgG (MSD; VIS 86, p = 0.0159, Supplementary Fig. 18), SARS-CoV-2 nucleocapsid IgG (MSD; VIS 67, p = 0.0328, Supplementary Fig. 18). A high frequency of CD14^hi^CD16^hi^ intermediate monocytes and granulocytes two weeks post-V1 also predicted high throat viral load (both VIS 59). A high abundance of CD14^hi^CD16^hi^ intermediate monocytes in peripheral blood significantly differentiated high from low throat viral loads (p = 0.006, Fig. 5c and Supplementary Fig. 18).

The abundance of several different T cell subsets throughout the vaccination regimen predicted low throat viral load (Fig. 5c). A higher abundance of CD4^+^CD95^+^ T cells (VIS 66, p = 0.0103, Fig. 5c and Supplementary Fig. 18), CD8^+^PD1^+^ T cells (VIS 62, p = 0.0172, Fig. 5c and Supplementary Fig. 18), CD4^+^PD1^+^ T cells (VIS 55, p = 0.0397, Fig. 5c and Supplementary Fig. 18), CD4^+^ T cells (VIS 51, p = 0.0283, Fig. 5c and Supplementary Fig. 18) and CD3^+^ T cells (VIS 51, p = 0.0279, Fig. 5c and Supplementary Fig. 18) at baseline significantly differentiated low from high throat viral load groups and could predict throat viral control. A higher frequency of peripheral CD3^+^ T cell two weeks post-V2 (VIS 57, p = 0.0222, Fig. 5c and Supplementary Fig. 18) and CD8^+^PD1^+^ T cell four weeks post-V1 (VIS 53, p = 0.0291, Fig. 5c and Supplementary Fig. 18) also predicted low throat viral load outcomes post-challenge.

### Immune features that predict the emergence of the protective SARS-CoV-2-specific humoral response

As spike-binding antibody was a consistently strong predictor of clinical and viral protection, SIMON ML analysis was performed to identify the early immune parameters that predict the emergence of high or low spike binding IgG titres in the vaccinated cohort only. Pre-immunisation serology (referred to as baseline serology from now on) was included in this analysis to investigate the phenomenon of original antigenic sin (OAS) in the context of SARS-CoV-2. Potential macaque seropositivity to human seasonal HCoVs was observed (Supplementary Fig. 2), as has been reported elsewhere^52^.

**Fig. 6:**
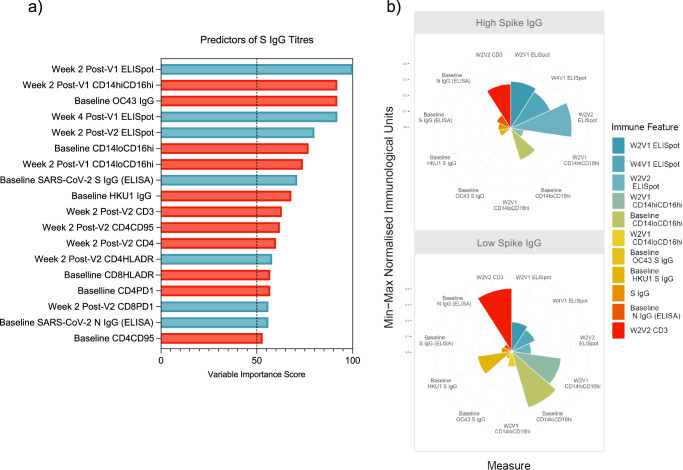
Immune predictors of the magnitude of the spike IgG response post-immunisation. Predicting magnitude of spike IgG responses (**a**) Variables of importance for spike IgG titres–predictors of high spike IgG titresin blue and predictors of low spike IgG titres in red. **b** Polar plot showing the difference in magnitude of the immune responses that predict high or low spike IgG titres.

The best performing model was built using weighted subspace random forest (wsrf) algorithm (train AUROC 0.775 and test AUROC 0.8333, Supplementary Fig. 12) which used 18 immune features to predict spike IgG titres (VIS > 50, Fig. 6a).

High baseline IgG responses to seasonal beta-HCoV spike predicted low SARS-CoV-2 spike IgG titres; HCoV-OC43 (MSD; VIS 92, p = 0.008, Fig. 6b, Supplementary Fig. 20) and HCoV-HKU1 (MSD; VIS 68, p = 0.0183, Fig. 6b, Supplementary Fig. 20). However, high SARS-CoV-2 spike IgG or nucleocapsid IgG responses at baseline predict high spike IgG titres post-vaccination (VIS 71 and 56, respectively), although differences between the response groups were insignificant.

In line with parameters that predicted pathology outcomes and high throat viral loads, high intermediate and non-classical monocyte frequency two weeks post-V1 also significantly predicted low spike IgG titres; CD14^hi^CD16^hi^ (VIS 92, p = 0.0085, Fig. 6b, Supplementary Fig. 20) and CD14^lo^CD16^hi^ (VIS 74, p = 0.0025, Fig. 6b, Supplementary Fig. 20). A high frequency of non-classical monocytes at baseline also predicted low spike IgG titres (VIS 77) although there was an insignificant difference in frequency between the low and high responder groups (Fig. 6b, Supplementary Fig. 20).

SARS-CoV-2-reactive T cells measured by IFNγ ELISpot, two weeks post-V1 (VIS 100), four weeks post-V1 (VIS 92) and two weeks post-V2 (VIS 80) positively predicted high spike IgG titres in serum. IFNγ ELISpot two weeks post-V1 had a VIS of 100 and significantly differentiated the high from low spike IgG titre groups (p = 0.0219, Fig. 6b, Supplementary Fig. 20). Two weeks post-V2 IFNγ ELISpot had a VIS of 80 and also significantly differentiated the high from low spike IgG titre groups (p = 0.0058, Fig. 6b, Supplementary Fig. 20), providing evidence for the importance of the T cell response in supporting antibody development. Additionally, SARS-CoV-2-reactive T cells measured by IFNγ ELISpot, both two weeks post-V1 and -V2, positively correlated with binding antibody titres on the day of challenge with statistical significance (r = 0.5125, p = 0.0002, and r = 0.5411, p = 0.002, respectively, Supplementary Fig. 21a).

The potential for outlying IFNγ ELISpot and CD14^lo^CD16^hi^ monocyte data measured during the immunisation regimen to contribute to their identification as predictors of spike IgG titres by the wsrf algorithm, was discredited by time-course plots (Supplementary Fig. 22).

A high frequency of T cell subsets, including CD3^+^, CD4^+^ CD95^+^ and CD4^+^ cells, in the periphery, two weeks post-V2 predicted low spike IgG titres (VIS 63, 62 and 60, respectively). High CD4^+^PD1^+^ (VIS 57) and CD4^+^CD95^+^ (VIS 53) T cell frequency at baseline, also predicted low spike IgG titres. However, high CD4^+^PD1^+^ and CD4^+^CD95^+^ T cell frequency at baseline and CD3^+^ T cell frequency at two weeks post-V2 also predicted low throat virus. Therefore, mature spike-reactive T cells may mediate viral control independent of their role in supporting antibody development. We found a significant positive correlation between the peripheral concentration of CD8^+^CD95^+^ cells on the day post-challenge and the rate of viral clearance in the URT of vaccinated macaques that experienced peak throat viral load on the day post-challenge (n = 10, Spearman correlation, r = 0.8024, p = 0.0074, Supplementary Fig. 21b). Although, the spike-reactivity of this CD8^+^ T cell population cannot be confirmed, the fact that the correlation was lost when the CD8^+^CD95^+^ frequency data for all macaques (including unvaccinated macaques) were included, suggests that this was a reactivated vaccine-induced memory CD8^+^ T cell response which contributed to the viral control post-challenge. In addition to our SIMON analysis that revealed that CD3^+^ T cell frequency two weeks post-V2 and CD8^+^PD1^+^ T cell frequency four weeks post-V1 predicted low throat virus post-challenge, this finding provides additional support for a role for T cells in viral control that is independent of the humoral response.

## Discussion

Vaccination aims to achieve either protection from severe clinical disease or reduced transmission, with optimal immunisation strategies successfully reaching both endpoints. Although several SARS-CoV-2 vaccines have been authorised for use, a definitive CoP for SARS-CoV-2 has not yet been defined. Additionally, it is uncertain whether current vaccines limit transmission. Here, we used a systems immunology approach to analyse pre-clinical data from NHP challenge studies.

Using integrative analysis and predictive modelling, we show that peripheral spike-binding IgG and NAbs are strong predictors of clinical protection. Spike-binding IgG and NAbs may therefore be a species- and vaccine-independent CoP for SARS-CoV-2. We demonstrate that binding and neutralising Abs together predict clinical protection, fulfilling the optimal conditions of a CoP that is also readily measurable in serum. Our results add to the body of evidence from human population-level studies that binding and neutralising antibodies are CoPs for SARS-CoV-2^9–15^. Earle et al. also drew strong significant correlations between NAbs and binding antibodies and vaccine efficacy, and Khoury et al. successfully modelled vaccine efficacy using NAb titres from seven phase III vaccine trials (Pfizer, Moderna, Gamaleya, AstraZeneca, Sinovac, Novavax, and Johnson & Johnson)^16,17^. Benkeser et al. concluded that differences in vaccine efficacy could be accounted for by variations in binding antibody and NAb titres induced by different vaccine platforms^13^. Therapeutic and prophylactic administration of convalescent serum or mAbs in humans and preclinical models also provides strong evidence that NAbs are a mechanistic CoP, protecting from severe disease^1–8^.

While clinical protection was best predicted by high NAb titres, we found that low viral loads in the sampled throat and lung was best predicted by high spike-binding IgG titres. Peripheral NAb titres had lower variable of importance scores in our lung viral load and throat viral load models and were poorer predictors of viral control. Earle et al. also proposed that the relationship between vaccine efficacy was strongest with binding antibody titres rather than NAb titres^16^. Gilbert et al. reports that only 68% of human vaccine efficacy was explained by peripheral NAbs^11^. Therefore, spike-binding IgG titres may be a more reliable correlate of both clinical and viral load protection outcomes in the context of SARS-CoV-2.

As the humoral response is critical for viral and clinical protection, it is important to understand the factors that impact the emergence of this response, particularly to support the design of future vaccines. We used SIMON to identify algorithms that model SARS-CoV-2 spike IgG titres on the day of challenge and we found that baseline responses to OC43 and HKU1 predicted low SARS-CoV-2 spike IgG titres. An explanation for this may be original antigenic sin (OAS), a phenomenon whereby memory B cells, reactive to seasonal CoVs, are reactivated in response to a related antigen exposure, and secrete antibody against the historical epitope, *in lieu* of the development of a naïve B cell response, mounted specifically against the new strain of the pathogen. Schiepers et al. described a ‘molecular fate mapping’ experiment in the context of SARS-CoV-2, and reported that 73% of anti-BA.1 antibodies following a heterologous BA.1 boost, were in fact derived from wild-type (WT)-memory B cells, and targeted conserved RBD epitopes between WT and BA.1, reflective of OAS^53^. We also report that HKU1 spike and SARS-CoV-2 nucleocapsid binding antibody at baseline are predictive of high viral load in the throat. In NHPs with higher seasonal CoV serology, the boosting of a cross-reactive antibody response may limit the magnitude of the humoral response targeting SARS-CoV-2 spike epitopes, and therefore increase the SARS-CoV-2 burden upon challenge.

The IgG subclass profile was of significant importance for predicting clinical outcomes and BAL viral load post-challenge. Binding antibodies that mediate Fc-effector functions can collaborate with innate cells to destroy SARS-CoV-2-infected cells, thus providing an explanation for the IgG subclass role in viral control. All four IgG subclasses emerged as predictors of clinical protection and low lung viral load. Macaque IgG1 has the greatest affinity of the IgG subclasses for activating FcγRIIa and FcγRIIIa^54,55^. However, as IgG2, IgG3 and IgG4 profiles were also returned as VOIs in the model for lung viral load and clinical protection, it is possible that a multi-subclass response is required to limit viral burden and pathology. Multi-subclass spike IgG responses and Fc-mediated effector functions also underpinned complete protection of RhMs vaccinated with two doses of NVX-CoV2373^56^. The functionality of antibody in the immunised macaques in our study is under further investigation. Spike-binding IgM was also identified as a predictor of clinical protection, lung viral load and throat viral load. SARS-CoV-2 IgM has been reported to be elevated in children, which coincided with stronger Fc responses^57^. As children experience mild or subclinical COVID-19, it is possible that the IgM response, with greater breadth and avidity, is mediating this protection.

We found that a high peripheral CD14^hi^CD16^hi^ intermediate monocyte frequency predicted pathology and high throat viral load outcomes. Delorey et al. similarly reported on the negative involvement of this monocyte population in the autopsies of 17 human patients who succumbed to SARS-CoV-2 infection. They reported enrichment of SARS-CoV-2 RNA in CD14^hi^CD16^hi^ monocytes, specifically, as well as notable CD16^+^ monocyte transcriptional changes in patients with severe pathology^58^. Koutsakos et al. also reported an association between CD16^+^ monocytes and severe COVID-19 cases requiring intensive care^59^. These observations may be explained by CD16-mediated uptake of SARS-CoV-2 initiating pro-inflammatory pyroptosis^60,61^. However, we also found that a high frequency of peripheral CD14^hi^CD16^hi^ intermediate monocytes early during the immunisation regimen, predicted low spike IgG titres. This is in line with reports that the IFNγ response to a *Salmonella typhimirium* co-infection with influenza in mice promotes monocyte recruitment that dysregulates germinal centre (GC) B cell metabolism to restrict the development of the humoral response to influenza^62^. Similar observations have been made for *Listeria monocytogenes*, Lymphocytic choriomeningitis virus and Chikungunya viral infections^62–64^. Therefore, identification of intermediate monocyte frequency as a predictor of pathology and high throat viral load outcomes, may be explained by intermediate monocyte-mediated restriction of the development of the protective humoral response in the GC.

T cells likely mediate protection from SARS-CoV-2, as asymptomatic cases of COVID-19 without seroconversion occur, and patients with immunoglobulin deficiency are protected against COVID-19, with both scenarios documenting T cell responses^65–72^. However, identifying a mechanism of T cell-mediated protection, that is exclusive of the role of T cells in supporting antibody development in seropositive individuals, is a challenge. This provides an explanation for the lack of T cell CoP studies, in comparison to the abundance of antibody CoP studies. The lack of standardised T cell assays and the lack of agreeance between the T cell assays in use, also contribute^73^.

Successful attempts have been made to demonstrate a relationship between T cells and viral control in pre-clinical models. Hasenkrug et al. found that CD4^+^ and CD8^+^ depletion prior to challenge delayed infection resolution by seven days in comparison to immunologically in-tact RhMs^74^. Depletion of CD8^+^ T cells in Syrian hamsters and K18-hACE2 mice abolished the viral control capacity achieved by treatment with a functional Fc mAb^75^. McMahan et al. depleted CD8^+^ T cells from RhMs following primary challenge and prior to re-challenge, to ascertain the role of memory SARS-CoV-2 reactive T cells in a realistic immunisation setting. In support of our findings that post-vaccination T cell abundance predicts low throat viral load, 100% of CD8^+^ T cell-depleted RhMs, versus 20% of control RhMs, had detectable SARS-CoV-2 vRNA in nasal swabs^76^. Th1 and Tfh cells were predictive of viral load in the nose, independently of antibody, in mRNA-173 vaccinated RhMs^2^ and CD8^+^ T cells, induced following intranasal vaccination of CyMs, also correlated with viral load in the nose independently of NAb titres^77^. Both CD4^+^ and CD8^+^ T cells significantly inversely correlated with replicating viral titres in the nose and throat in the macaque model of Omicron infection^78^.

Another approach to carrying out T cell CoP studies is to track the magnitude and timing of a recall T cell response following breakthrough infection. This was performed in human vaccinees by Koutsakos et al. who reported that upon breakthrough infection, spike-specific CD8^+^ T cells became rapidly activated and cytotoxic^79^. This study demonstrated that the frequency of activated spike-specific CD38^+^CD8^+^ T cells during infection was positively correlated with viral decay rate and negatively correlated with peak viral load^79^. This was similarly seen in primary acute infection, where SARS-CoV-2 reactive T cells were associated with accelerated viral clearance^80^ and mild disease^81^.

Following SIMON analysis, we found that T cell frequency during the immunisation regimen, in particular a high peripheral CD8^+^PD1^+^ T cell frequency, predicted viral control. SARS-CoV-2-reactive T cells two weeks post-V1 also predicted low lung viral load and high spike IgG titres. We suggest that peripheral T cell frequencies and SARS-CoV-2 T cell reactivity predicted challenge outcomes at early timepoints post-vaccination, as they likely became memory T cells and retreated to secondary lymphoid organ memory compartments to come into effect when recalled upon challenge or, alternatively, became tissue-resident memory T cells, as observed in the lungs of mild COVID-19 patients^82^. We do report that SARS-CoV-2 reactive T cells are crucial for supporting antibody development. However, importantly, high peripheral T cell subset frequencies, particularly CD4^+^CD95^+^ T cells, predict protection and viral control, but predict low spike IgG titres, which may indicate a role for T cells in viral control that is independent of their role in humoral response development.

Limitations of this study include the lack of B cell data, as B cell markers were not included in the flow cytometry panels. Gagne et al. does report a role for memory B cells in mediating protection in RhMs challenged with the Delta strain of SARS-CoV-2 one year following mRNA-1273 vaccination, as anamnestic antibodies emerge four days post-challenge^83^. Additionally, mucosal and lung tissue data is lacking, which may explain our inability to model nasal viral load control. It is also worth mentioning that the only approved vaccine included in this study was the ChAdOx nCoV-19 vaccine, and only one dose of this vaccine was administered, rather than two-doses, as was approved. SARS-CoV-2-specific T cell responses induced by these vaccines were also generally low, thus bringing into question the use of the less sensitive IFNγ ELISpot assay for accurate assessment of the distribution of these low-end responses across our cohort (Ogbe et al. reports on the improved sensitivity of the proliferation assay^73^). Therefore, it is possible that the predictive power of T-cell responses has been downplayed in our analysis. More generally, we must appreciate the artificial nature of challenge trials that involve inoculation with large titres of virus to the respiratory mucosal site, which is not representative of true natural infection. The failure of macaque pre-clinical vaccine study results to translate to human clinical trials, as has been the case with HIV vaccine research^84^, must also be considered.

In conclusion, the working hypothesis is that NAbs effectively protect against clinical disease by limiting cell infection and the inflammatory response. However, peripheral NAbs alone cannot predict low viral load. We propose that T cells mediate viral control by killing any cell that becomes infected by SARS-CoV-2 that circumvents neutralistion, which may be particularly relevant in the context of circulating VOCs. Viral control may also rely on functional binding antibodies. Crucially, we found an incomplete overlap between immune parameters that predict clinical and viral protection. Particularly striking was the lack of agreement between the models on the importance of NAb titres. This highlights the requirement to define the term CoP more specifically, in relation to the outcome of interest post-immunisation. This may be protection from URT mucosal infection, severe clinical infection or limiting transmission potential. Vaccinologists may benefit from both a) a universal definition of the CoP for clinical protection and b) clear instruction from regulators that achieving protection from severe clinical infection is the priority in a pandemic setting. Once the disease is under control, as is the case for COVID-19, design of vaccines that also limit viral transmission may become the next aim, in which case a ‘correlate of transmission prevention’ should be drawn.

## Methods

### MesoScale Discovery Assay to Evaluate Baseline HCoV Antibody Responses Post-Vaccination/Challenge Breadth of Antibody Responses of HCoVs

MesoScale Discovery’s (MSD®, Rockville, MD) human coronavirus (HCoV)-antigen multiplexed-immunoplate, V-PLEX Coronavirus Plate 3 (K15399U), was coated with SARS-CoV-2 spike, RBD and nucleocapsid, SARS-CoV-1 and MERS-CoV spike trimers, spike from seasonal beta-coronaviruses, HCoV-OC43 and HCoV-HKU1, and seasonal alpha-coronaviruses, HCoV-NL63 and HCoV-229E, and Bovine Serum Albumin (BSA) at a concentration of 200-400 ug/ml. MSD® assays were performed as per the instructions of the manufacturer. Serum samples were diluted to 1:500 and 1:5000 in Diluent 100 and transferred to the plates (50ul), along with a 1:4 dilution series of Reference Standard 1 in Diluent 100, a blank (Diluent 100) and controls 1-3 added at their stock concentration. Non-human primate sera from baseline, day of challenge and 6-8 days post-challenge were tested and data from these assays were included in SIMON analysis.

### Serum Antibody Isotyping

A multiplex assay for macaque serum Ig isotyping was developed based on a protocol previously described in Brown et al.^85^ and applied to assess anti-EBOV Ig isotype by Koch et al.^86^. SARS-CoV-2 spike (1.53 mg/ml Wuhan strain stock, LakePharma) was covalently conjugated to carboxylated paramagnetic 4-4.9μm microspheres in the SPHERO™ Magnetic Blue Particle Array kit (Spherotech) containing beads of six different APC fluorescence intensities to facilitate multiplexing. A known SARS-CoV-2 convalescent plasma sample was used to confirm successful spike conjugation to the beads upon detection of IgG spike binding following incubation with PE-conjugated anti-human IgG (Southern Biotech #9040-09). A known SARS-CoV-2 spike negative sample (pre-pandemic serum from 2016) and a mouse IgG isotype control (BioLegend #400112) was used to confirm antibody binding specificity.

Each bead population was diluted to a concentration of 200 beads/μl in an assay buffer of PBS and 0.1% BSA and 50 μl was transferred to a flat-bottom 96-well plate (ThermoScientific™). Serum samples diluted 1:100 in assay buffer were transferred to their corresponding wells (50 μl). Plates were incubated for 2-hours in the dark at room temperature on a plate shaker at 540 rpm. Magnetic beads were retained on the Magnum FLX® plate magnet (Alpaqua #A00400) and supernatant discarded. Following a wash step and resuspension in assay buffer, the bead-bound Ig was incubated for one hour with 100 μl of 0.65 μg/ml of isotype-specific secondary antibodies (concentration used by^49,55,76^); mouse anti-monkey IgG-PE (SB108a, Southern Biotech #4700-09), mouse anti-rhesus IgG1 (3C10.3, Absolute Antibodies #01623-4.0), mouse anti-rhesus IgG2 (8D11, Leinco Technologies #R1500), mouse anti-rhesus IgG3 (6F5, Leinco Technologies, #R1600), mouse anti-rhesus IgG4 (7A8, Absolute Antibodies #01625-1.1) and mouse anti-monkey IgM (2C11, Life Diagnostics #2C11-1-5). Plates were incubated for 1-hour in the dark at room temperature on a plate shaker at 540 rpm.

Following the wash step and resuspension in assay buffer, the plates were incubated for one hour with 112.5 μl of 0.5 μg/ml PE-conjugated tertiary goat anti-mouse IgG antibody (Southern Biotech #1030-09) (as cited in^49,55,76^). Following the wash step, the bead pellet was resuspended in 200 μl of storage buffer and 100 μl from the well of each isotype of the same sample was transferred to their respective FACS tubes for flow cytometry analysis. Included on the plate, was a blank to assess the background, an isotype control, a negative control serum (2014 macaque serum) and a positive control of pooled SARS-CoV-2 immunised macaques for quality control purposes. Data was acquired on the BD LSRFortessa X-20. QuantiBRITE™ PE Fluorescence Quantitation kit (BD Biosciences) was used to standardise the reporting of results as PE molecules/bead rather than MFI units. For each assay, 10,000 QuantiBRITE™ events were recorded using the same cytometer settings as had been used for serum sample analysis. Low, Low Medium, High Medium and High PE bead’s MFI correspond to a known number of PE molecules/bead, from which a linear log(MFI) v log(PE molecules/bead) graph was generated for standardisation of serum Ig results in PE molecules/bead. Data was analysed using FlowJo v10.9.0.

### Data pre-processing and integration of the datasets

Data integration follows the workflow of extract-transform-load (ETL). Much of the data for this analysis was from individual pre-clinical vaccine trials compiled by the UK Health Security Agency, with data from the aforementioned assays added also^36–40^. The dataset was formatted such that each parameter at each timepoint was put forward as a candidate immune predictor of protection for SIMON analysis. Immunogenicity data was plotted and multiple comparisons tests were performed using R statistical software v4.1.2.

Methods described in Tsang et al.^87^ were adapted for IgG subclass categorisation by titre and ratio. Antibody IgG subclass data was base transformed and titres were ‘binned’ into categories depending on whether they fell within or between the lower and/or upper 20th percentile of titres. Within these titre bins, further binning occurred on the basis of IgG subclass ratio falling within or between the upper and/or lower 20th percentile of ratios with respect to the other IgG subclasses, so as to categorise each IgG subclass result into one of nine categories (Supplementary Fig. 15). The categorised IgG subclass data was included for SIMON analysis. ChAdOx MNA NT_50_ values included in the dataset were predicted from PRNT assays. This prediction is based on the regression between MNA and PRNT determined by Bewley et al. which is log_10_(MNA) = 0.3487 + 0.891(log_10_(PRNT))^88^.

Pathology scores, used to determine clinical protection outcomes, were assigned to SARS-CoV-2-challenged lungs harvested post-cull by veterinary pathologist using the published scoring system^36^. In circumstances where pathology scoring was unavailable for the macaque, CT scores during acute COVID-19 were used to assess protection status (occurred in 14 cases). Macaques with pathology scores above the median pathology score (median=6.75, Supplementary Fig. 6) were considered to have less protection, and so represented the ‘pathology’ group, whilst those below the median pathology score represented the ‘protected’ group. Two-tailed spearman correlations between immune response and pathology were plotted using R statistical software v4.1.2. The density plots and Venn diagrams were generated using R statistical software v4.1.2.

The outcome of viral protection in the nose, throat and lung, was based on the quantification of SARS-CoV-2 nucleocapsid RNA by RT-qPCR in nasal washes, throat swabs and bronchiolar lavage (BAL) fluid respectively. Area under the curve (AUC) of the nasal and throat PCR results were calculated to broadly capture viral control at these sites during acute infection. The median of nasal PCR AUC (median=1.5 ×10^7^), throat PCR AUCs (median=590,000) and BAL PCR from 6-8 days post-challenge (median=120,000) (Supplementary Fig. 7–9) defined the cut-off for the high and low virus group. The density plots were generated using R statistical software v4.1.2. Macaques that lacked PCR data were excluded from the analysis (nine macaques for lung viral load analysis, none for nasal or throat viral load analysis).

Further pre-processing was performed by SIMON software including data centering via subtraction of the mean (centre), data scaling by dividing by the standard deviated (scale), imputation of missing values (medianImpute) and removal of features with zero or near-zero variance (zv and nzv). Correlation filtering (corr) was not performed as a) immune features commonly correlate and b) all immunological measurements pre-challenge were included in the dataset, and so inter-timepoint correlations would exist (see Supplementary Fig. 21).

### Supervised machine learning using SIMON software for integrative analysis of immunological predictors of clinical and viral protection in macaques

To identify immunological signatures postimmunisation that universally predict clinical and/or viral protection irrespective of immunisation via infection or vaccination with any effective vaccine technology, we used machine learning (ML) platform SIMON (Sequential Iterative Modelling Over Night). 158 immune features from 77 macaques over the two-month vaccination and challenge regimen provided 12,166 datapoints to model ‘pathology’ or ‘protection’ outcomes post-SARS-CoV-2 challenge, with data sparsity of 44%. 158 immune features from 69 macaques over the two-month vaccination and challenge regimen provided 10,902 datapoints to model ‘low’ or ‘high’ lung viral load outcomes post-SARS-CoV-2 challenge, with data sparsity of 39%. 158 immune features from 90 macaques over the two-month vaccination and challenge regimen provided 14,220 datapoints to model ‘high’ or ‘low’ nasal and throat outcomes post-SARS-CoV-2 challenge, with data sparsity of 49%. 97 immune features from 48 vaccinated macaques over the two-month vaccination and challenge regimen provided 4,656 datapoints to model ‘high’ or ‘low’ spike IgG titres, with data sparsity of 41%. Data was partitioned 80% to the training set and 20% to the testing set, with the training set being used for model training and feature selection. The accuracy of feature selection by the models was determined by performing 10-fold cross-validation. Model performance was summarised by receiver operator curves (ROC) of sensitivity versus the false positive rate (1-specificity). Area under the ROC (AUROC) enables comparison of model performance. Model acceptance criteria is a train AUROC > 0.7, and a test AUROC>train AUROC (Supplementary Fig. 12).

The magnitude of the contribution of immune features to the building of the models was reported as a variable importance score within a range of 0-100. Immune features scored >50 were variables of importance and represent candidate predictors of protection.

The correlogram was generated using SIMON platform. Max-min normalised plots generated using R statistical software v4.1.2. Two-sided Mann Whitney statistical test performed and graphs generated using GraphPad Prism v9.3.1.

## Supplementary information

Supplementary information

Supplementary Data

## Data Availability

The raw data for all of the immune predictors identified by the SIMON analysis have been provided in the Supplementary Appendices. Any additional data will be made available on reasonable request to the corresponding author.

## Materials availability

All data used in this analysis will be provided in the Supplementary Information.
