# Supplementary material for: A systems biology approach to define SARS-CoV-2 correlates of protection
Source: NPJ Vaccines. 2025 Apr 14;10:69. doi: 10.1038/s41541-025-01103-2 (PMC11997207; doi:10.1038/s41541-025-01103-2)
Supplement: Supplementary file 1 — Supplementary information [file 41541_2025_1103_MOESM1_ESM.pptx]

## Slide 1
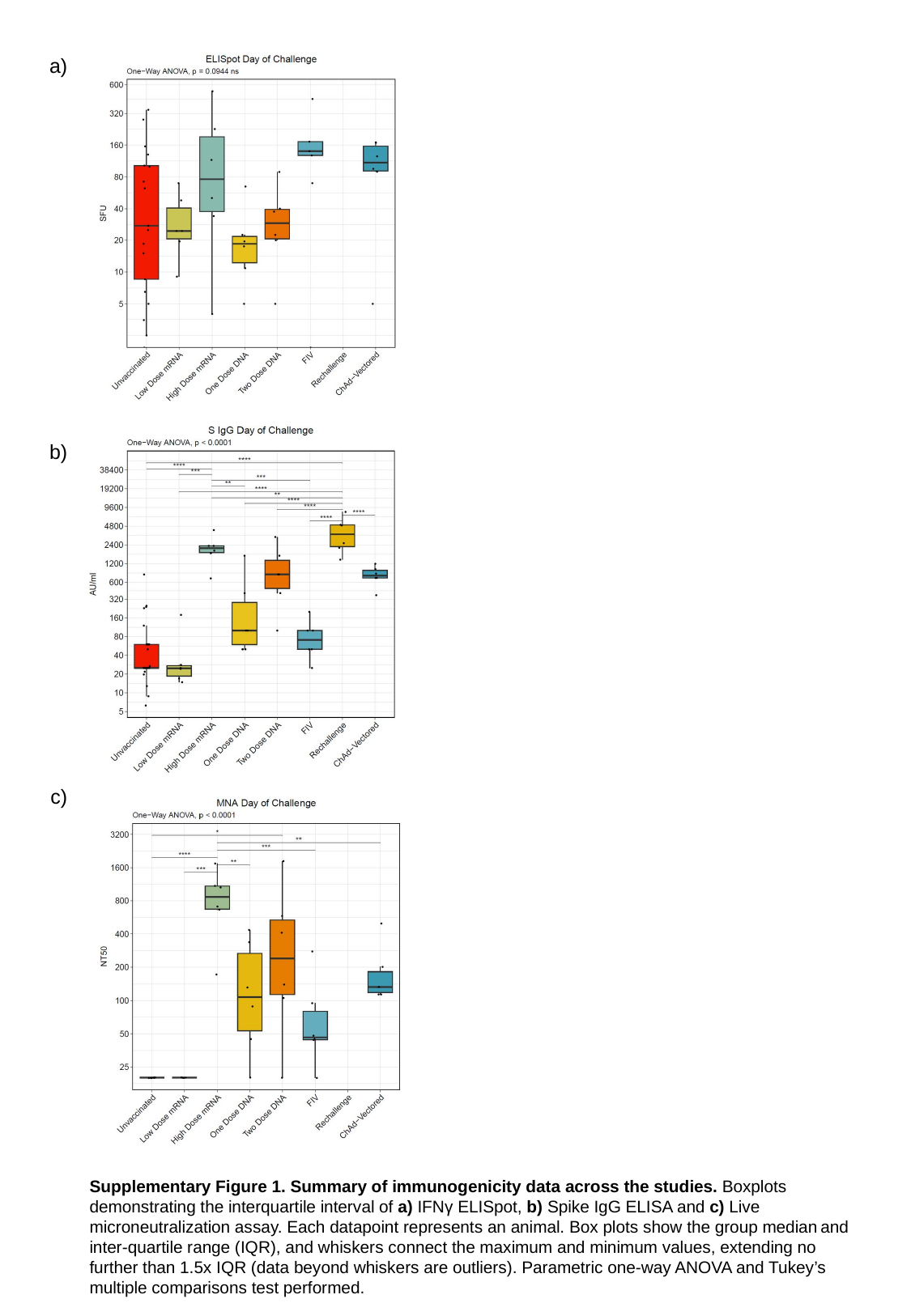

a)
b)
c)
Supplementary Figure 1. Summary of immunogenicity data across the studies. Boxplots demonstrating the interquartile interval of a) IFNγ ELISpot, b) Spike IgG ELISA and c) Live microneutralization assay. Each datapoint represents an animal. Box plots show the group median and inter-quartile range (IQR), and whiskers connect the maximum and minimum values, extending no further than 1.5x IQR (data beyond whiskers are outliers). Parametric one-way ANOVA and Tukey’s multiple comparisons test performed.

## Slide 2
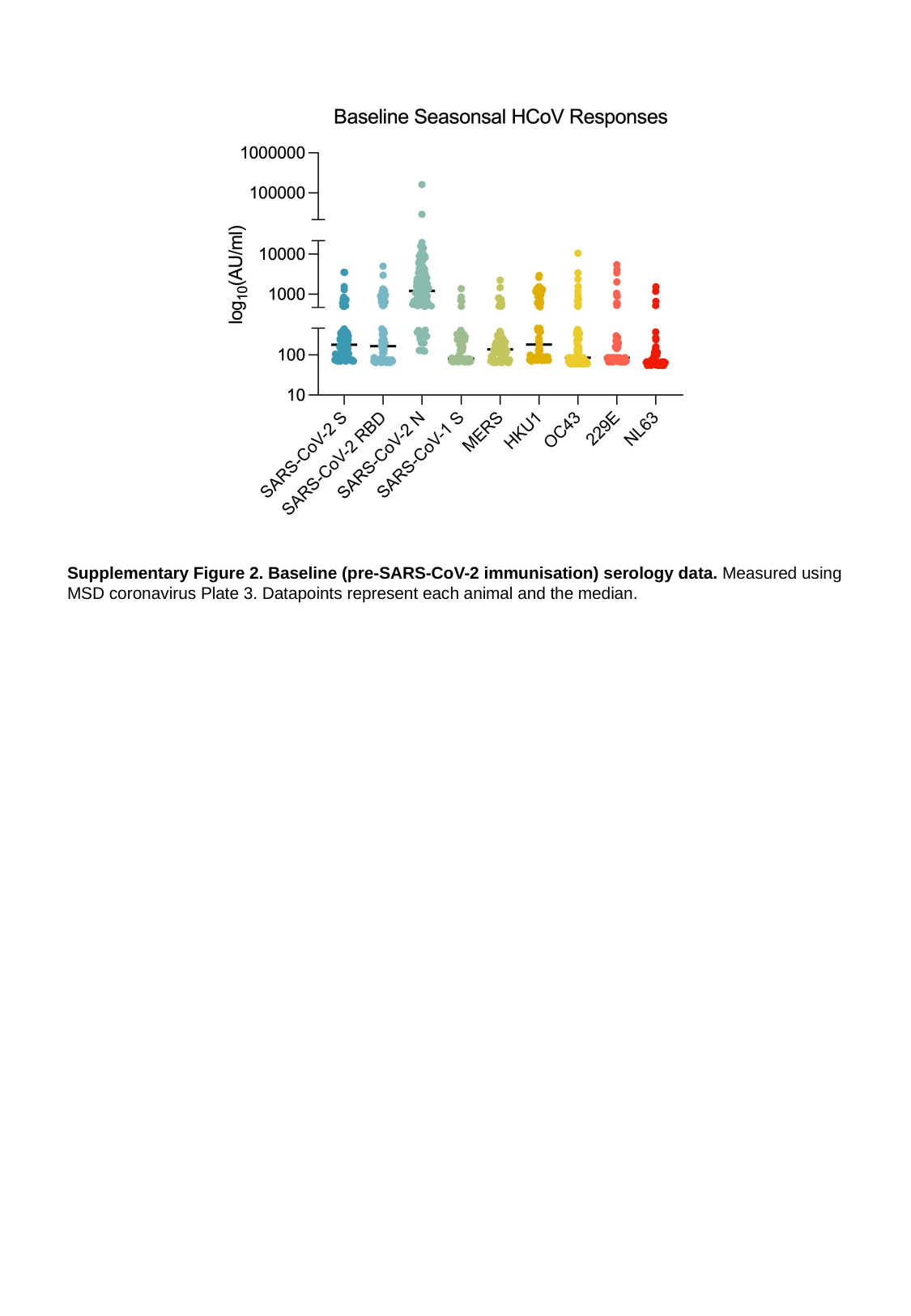

Supplementary Figure 2. Baseline (pre-SARS-CoV-2 immunisation) serology data. Measured using MSD coronavirus Plate 3. Datapoints represent each animal and the median.

## Slide 3
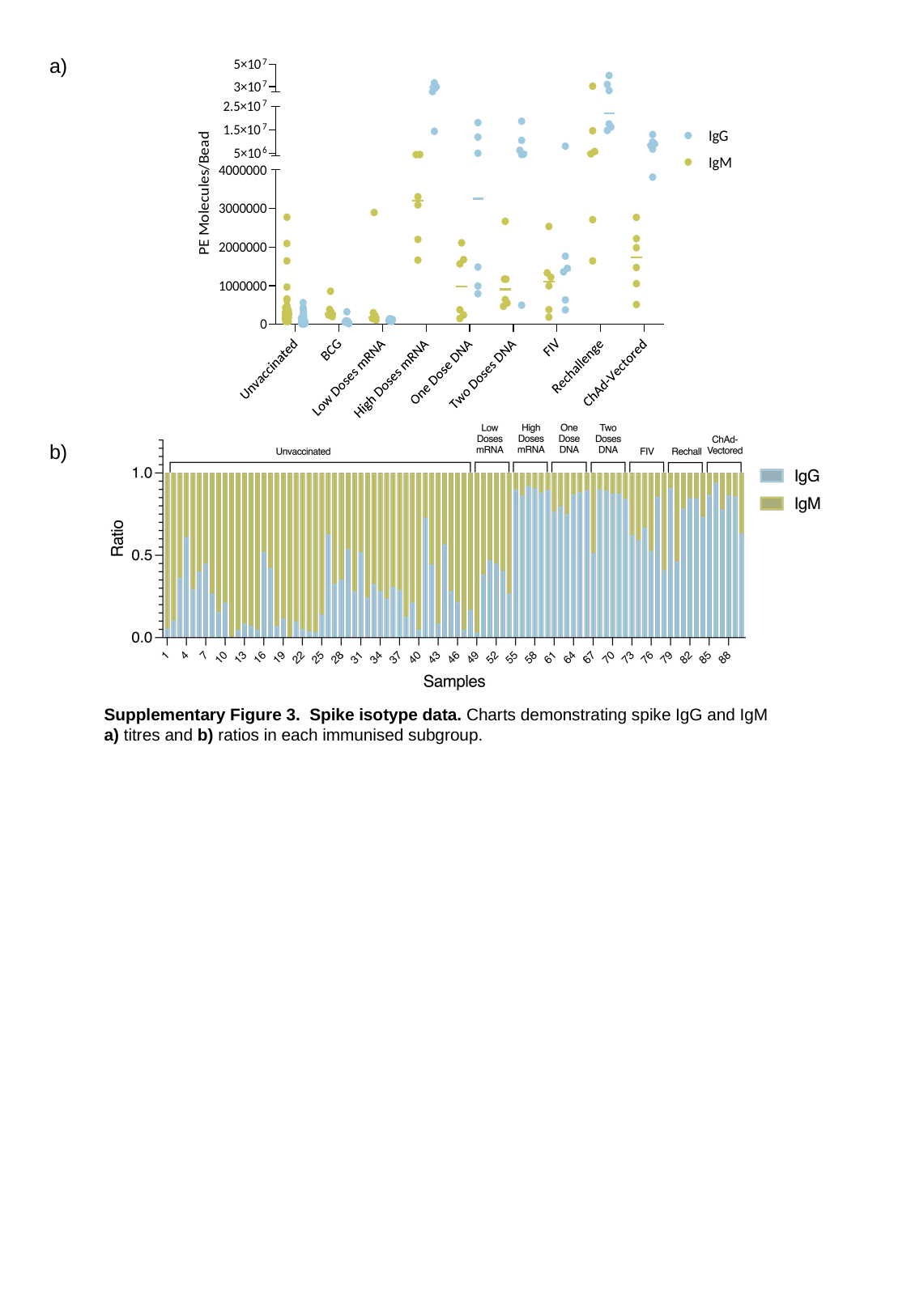

a)
b)
Supplementary Figure 3. Spike isotype data. Charts demonstrating spike IgG and IgM a) titres and b) ratios in each immunised subgroup.

## Slide 4
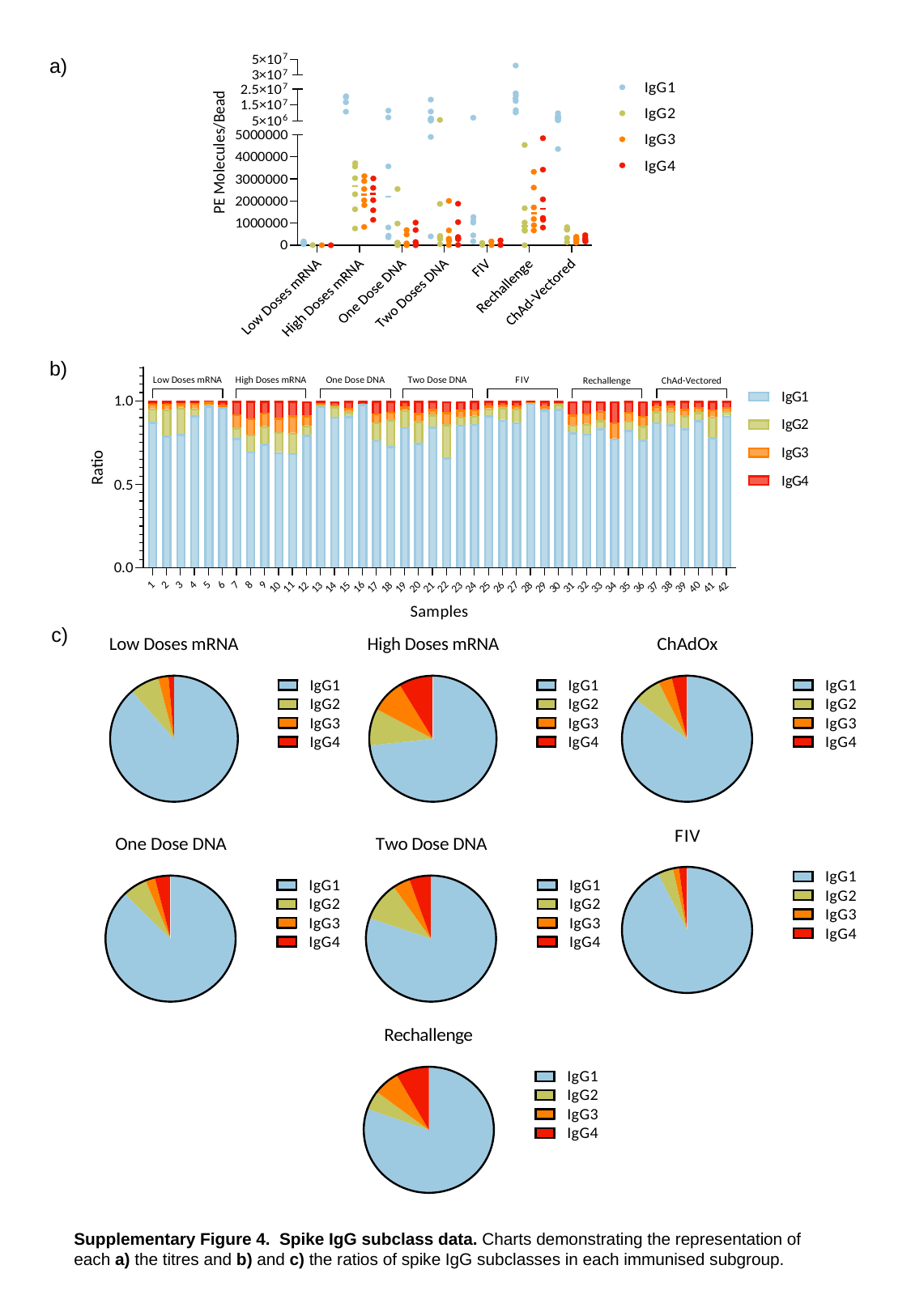

a)
b)
c)
Supplementary Figure 4. Spike IgG subclass data. Charts demonstrating the representation of each a) the titres and b) and c) the ratios of spike IgG subclasses in each immunised subgroup.

## Slide 5
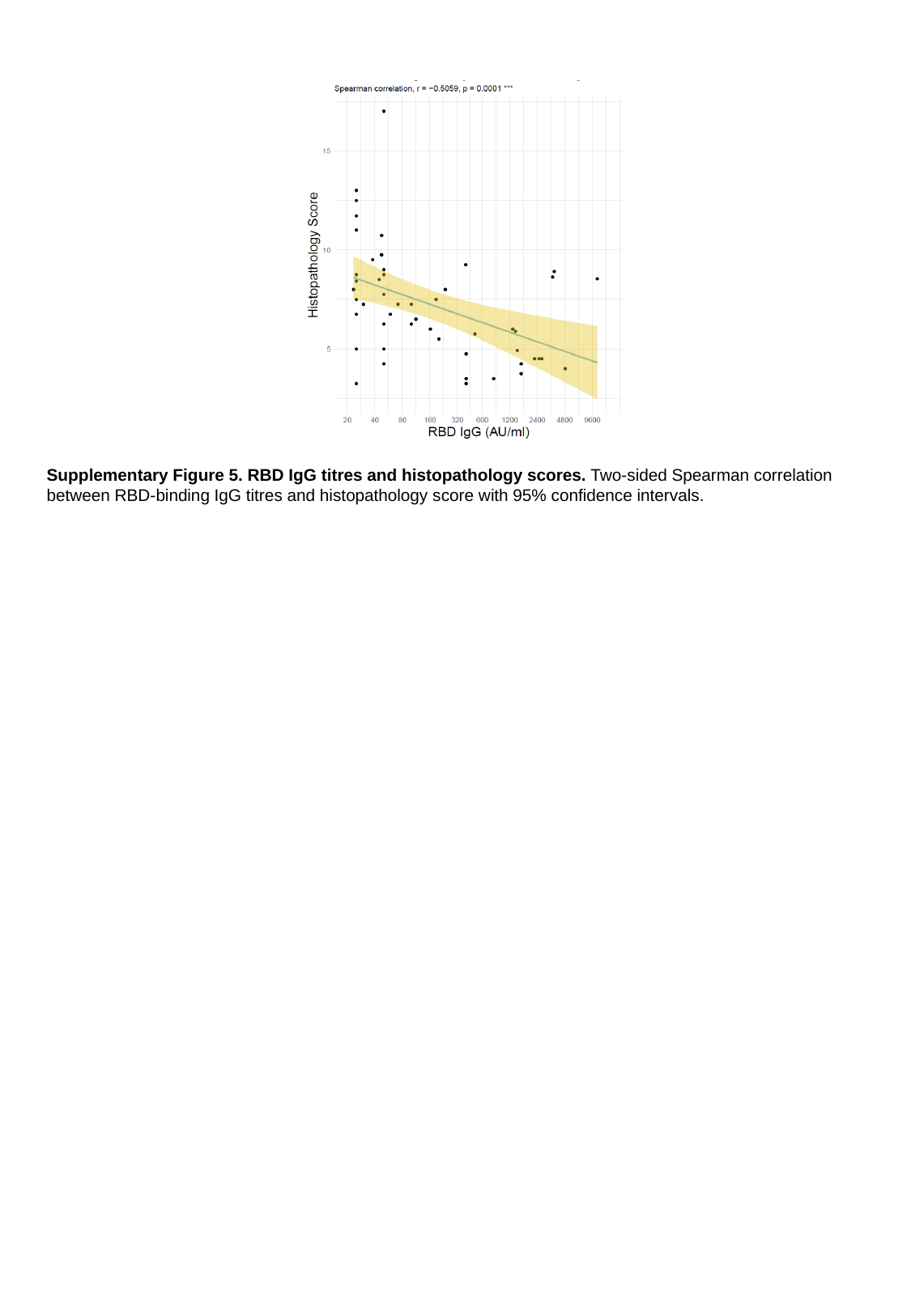

Supplementary Figure 5. RBD IgG titres and histopathology scores. Two-sided Spearman correlation between RBD-binding IgG titres and histopathology score with 95% confidence intervals.

## Slide 6
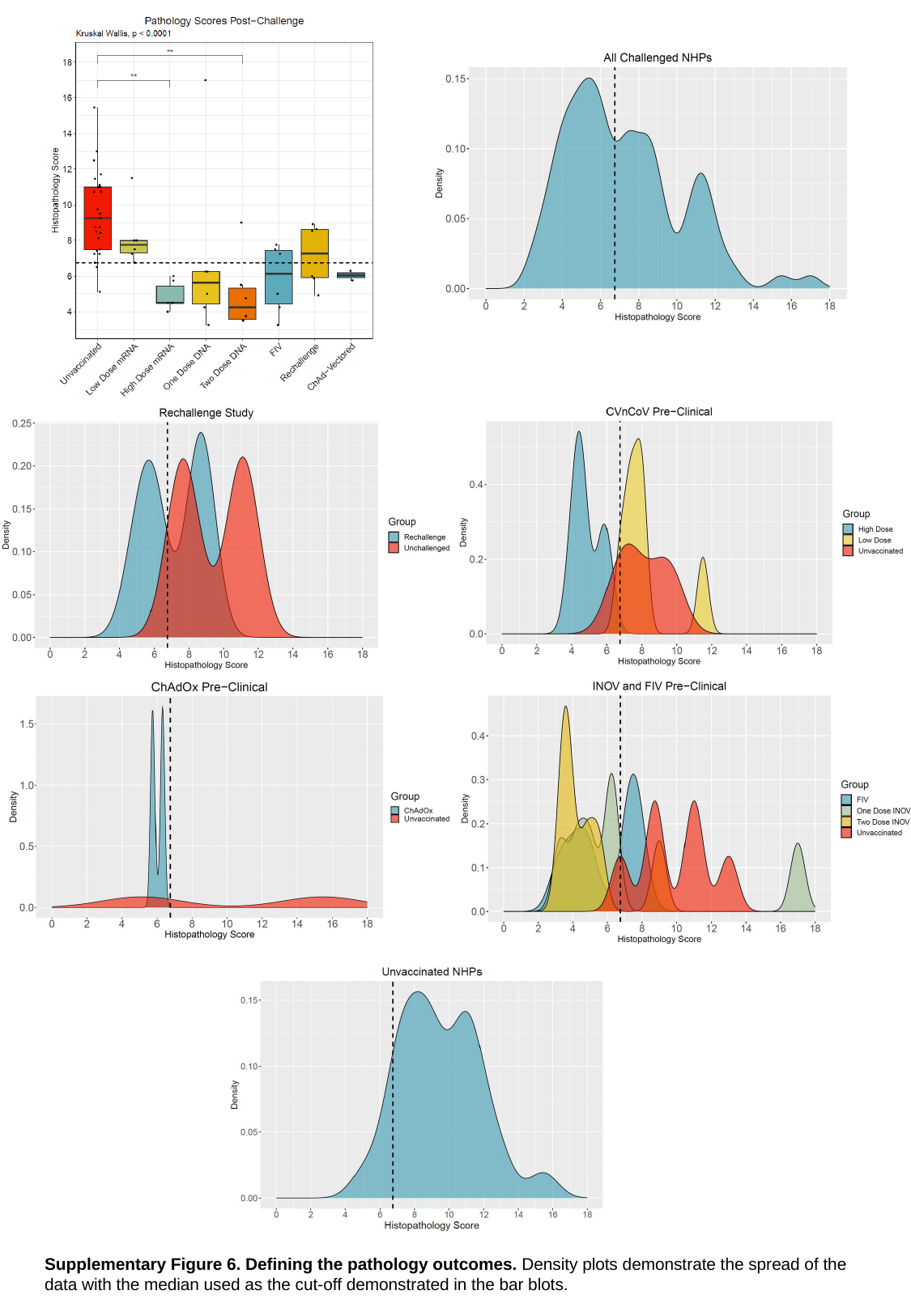

Supplementary Figure 6. Defining the pathology outcomes. Density plots demonstrate the spread of the data with the median used as the cut-off demonstrated in the bar blots.

## Slide 7
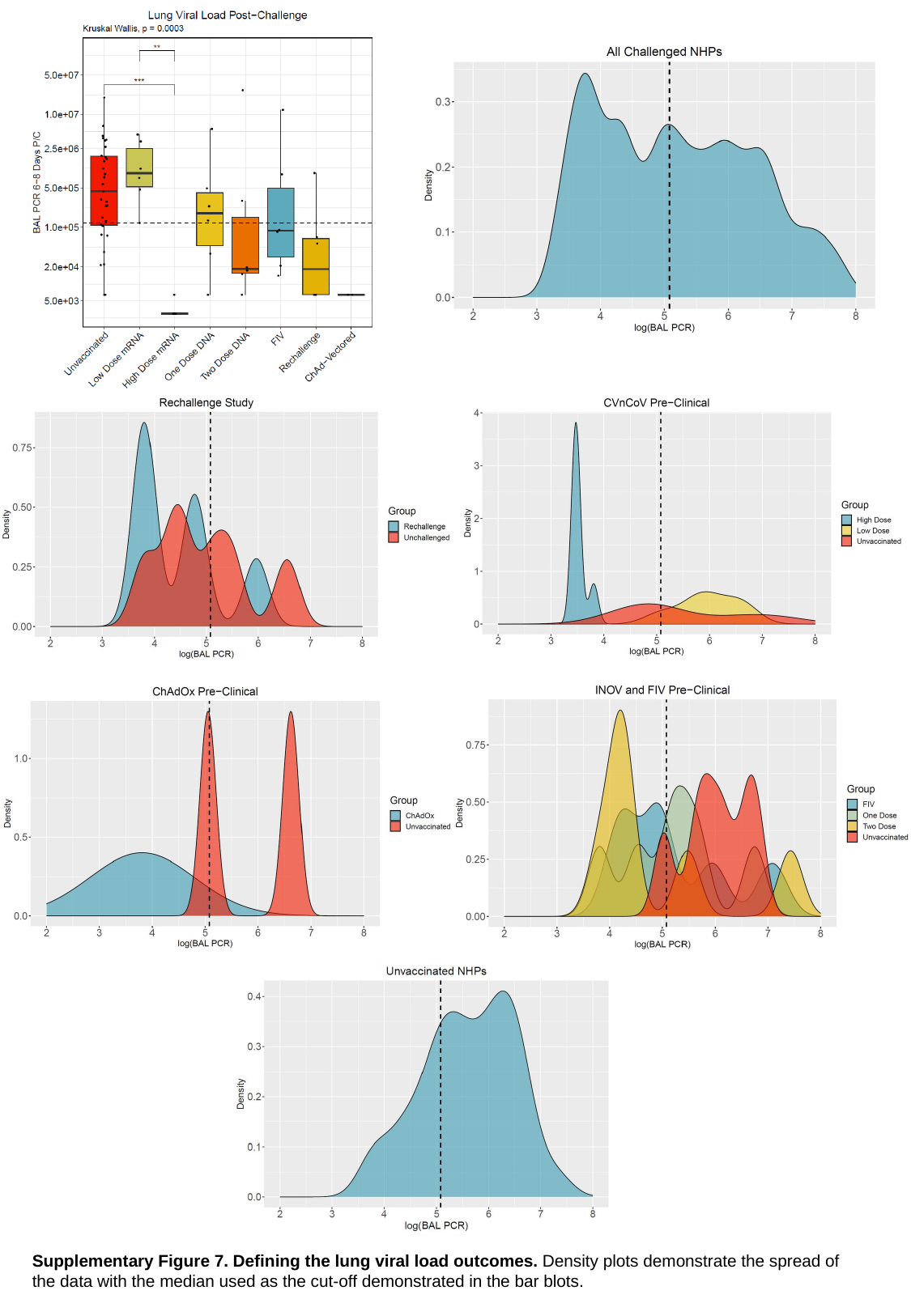

Supplementary Figure 7. Defining the lung viral load outcomes. Density plots demonstrate the spread of the data with the median used as the cut-off demonstrated in the bar blots.

## Slide 8
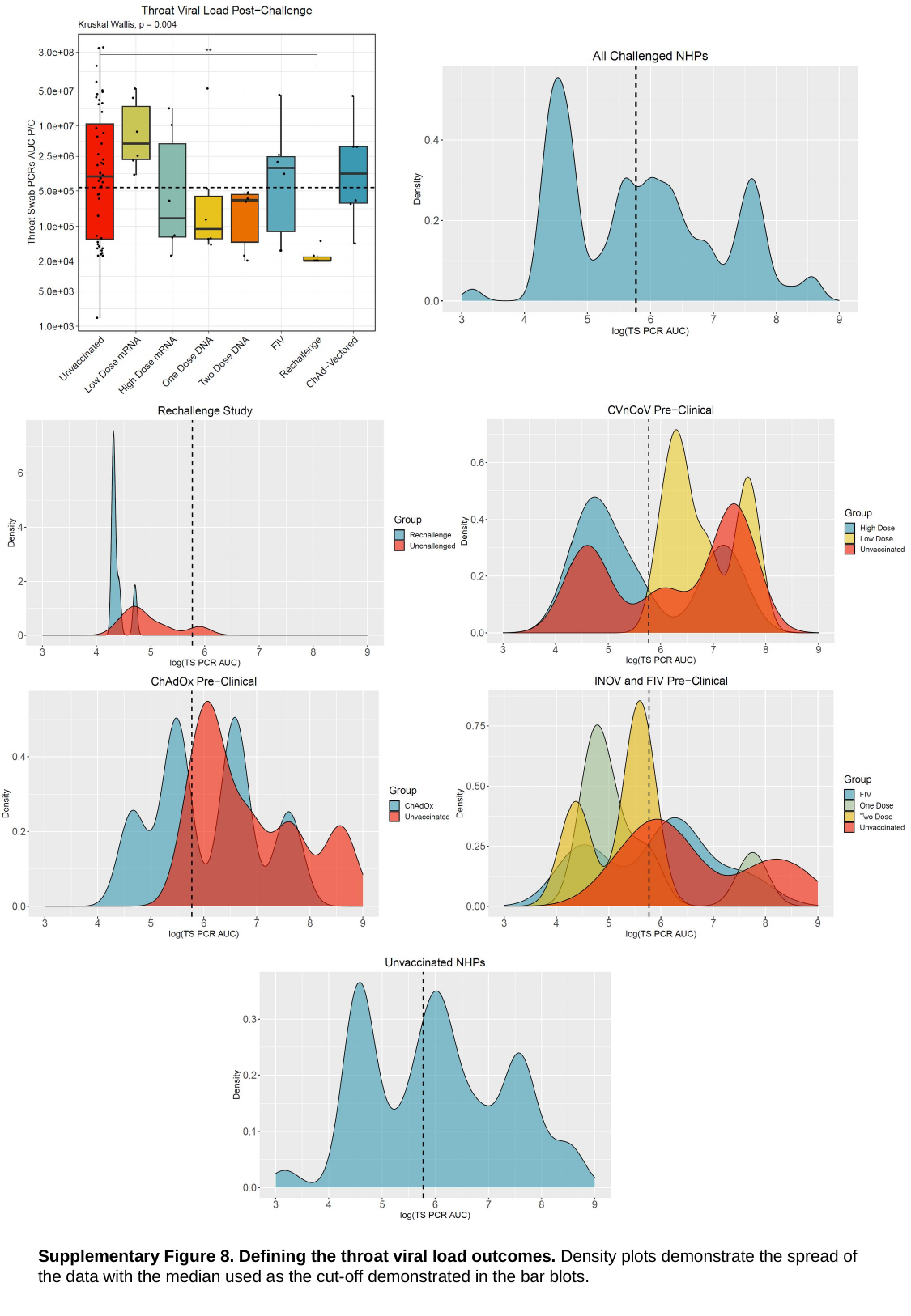

Supplementary Figure 8. Defining the throat viral load outcomes. Density plots demonstrate the spread of the data with the median used as the cut-off demonstrated in the bar blots.

## Slide 9
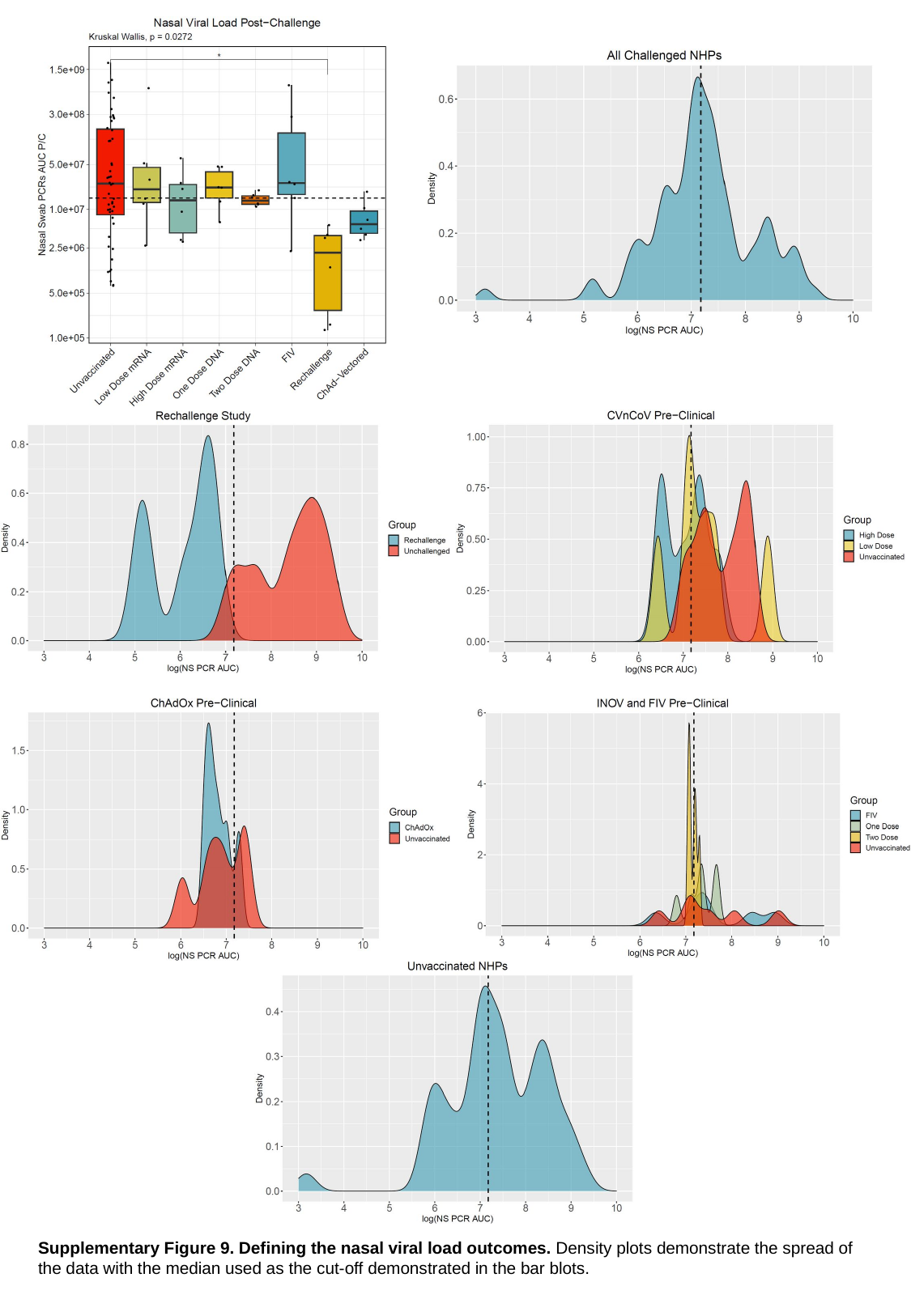

Supplementary Figure 9. Defining the nasal viral load outcomes. Density plots demonstrate the spread of the data with the median used as the cut-off demonstrated in the bar blots.

## Slide 10
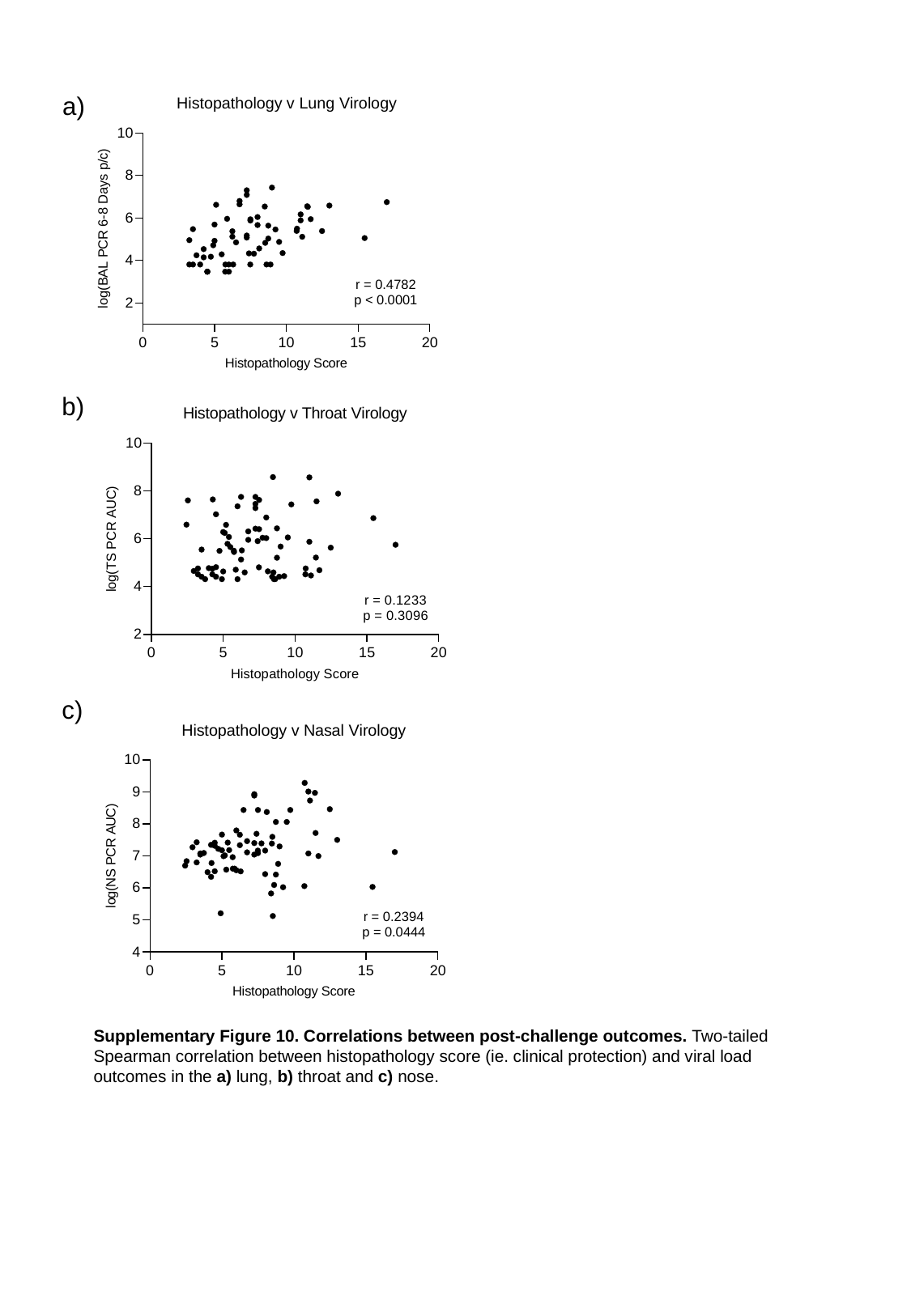

a)
b)
c)
Supplementary Figure 10. Correlations between post-challenge outcomes. Two-tailed Spearman correlation between histopathology score (ie. clinical protection) and viral load outcomes in the a) lung, b) throat and c) nose.

## Slide 11
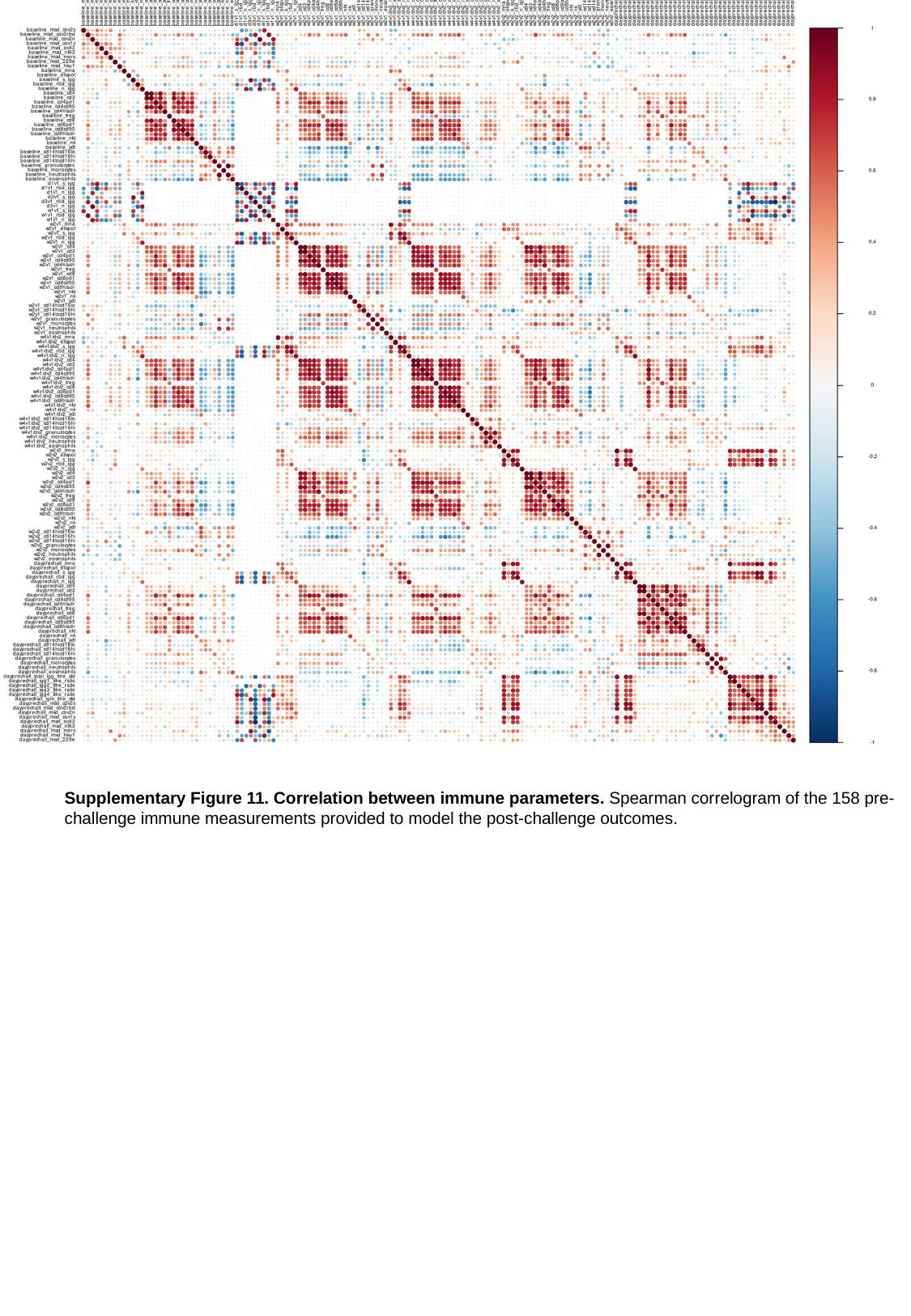

Supplementary Figure 11. Correlation between immune parameters. Spearman correlogram of the 158 pre-challenge immune measurements provided to model the post-challenge outcomes.

## Slide 12
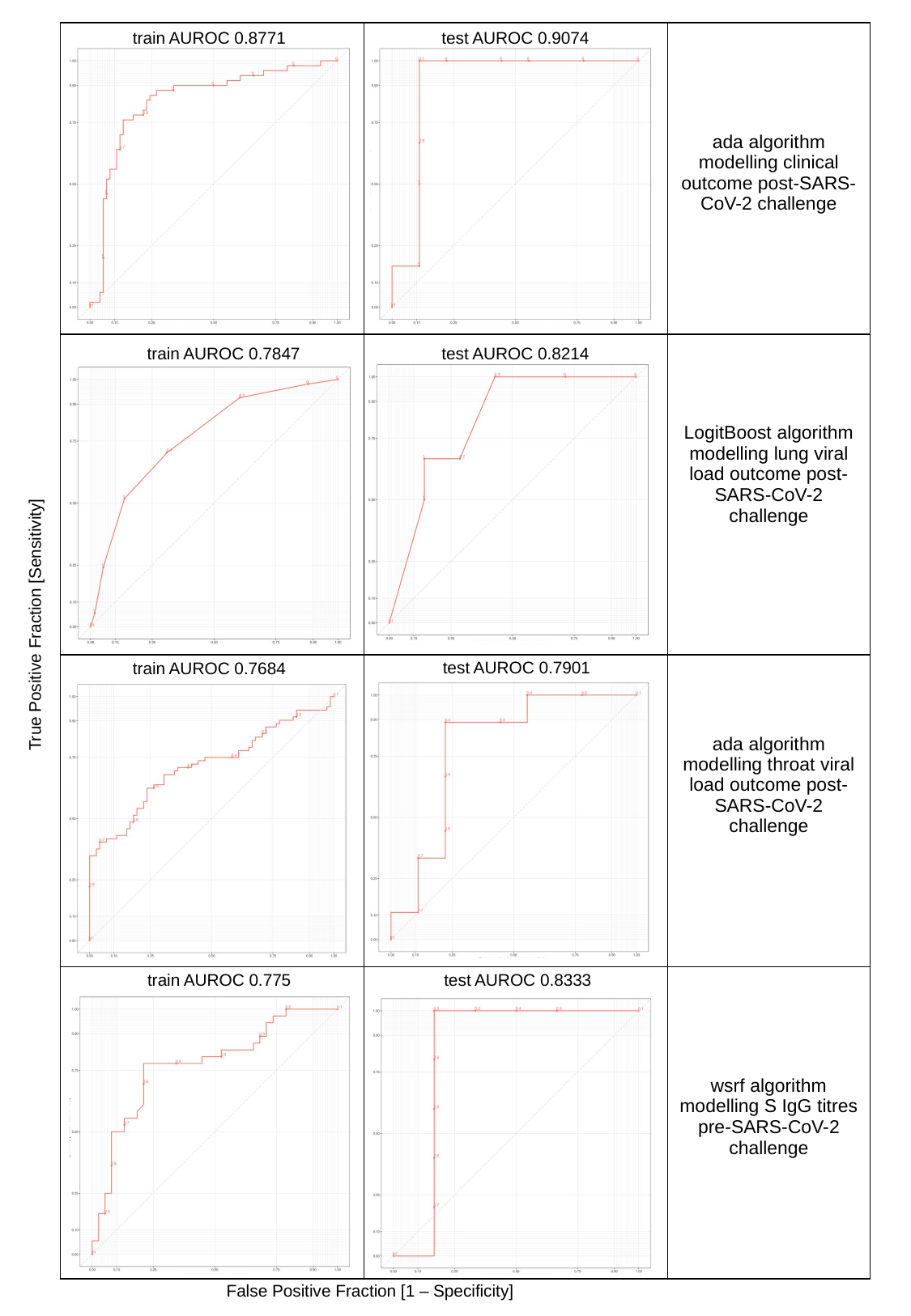

train AUROC 0.8771
test AUROC 0.9074
train AUROC 0.7847
test AUROC 0.8214
True Positive Fraction [Sensitivity]
False Positive Fraction [1 – Specificity]
| | | ada algorithm modelling clinical outcome post-SARS-CoV-2 challenge |
| --- | --- | --- |
| | | LogitBoost algorithm modelling lung viral load outcome post-SARS-CoV-2 challenge |
| | | ada algorithm modelling throat viral load outcome post-SARS-CoV-2 challenge |
| | | wsrf algorithm modelling S IgG titres pre-SARS-CoV-2 challenge |
test AUROC 0.7901
train AUROC 0.7684
train AUROC 0.775
test AUROC 0.8333

## Slide 13
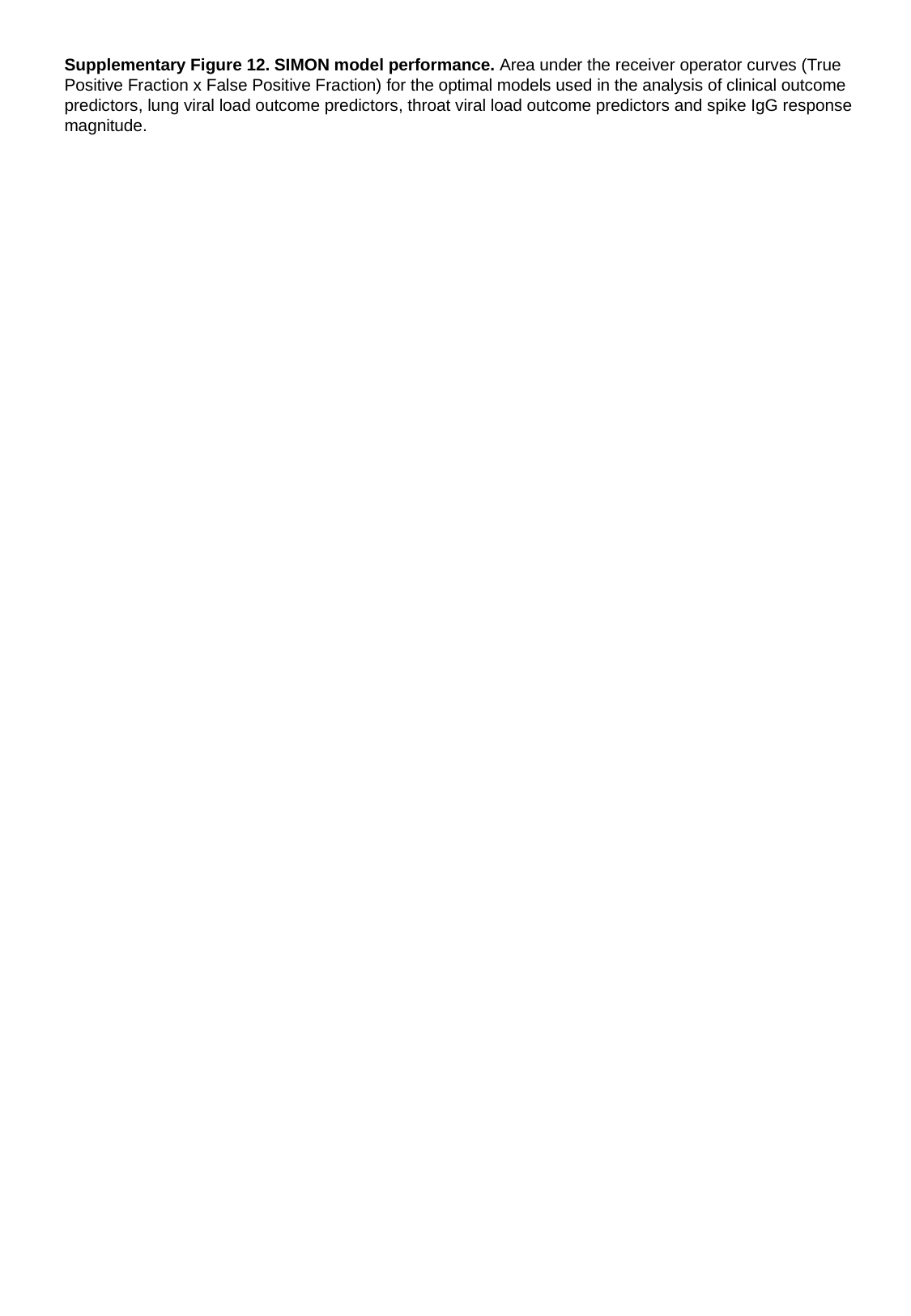

Supplementary Figure 12. SIMON model performance. Area under the receiver operator curves (True Positive Fraction x False Positive Fraction) for the optimal models used in the analysis of clinical outcome predictors, lung viral load outcome predictors, throat viral load outcome predictors and spike IgG response magnitude.

## Slide 14
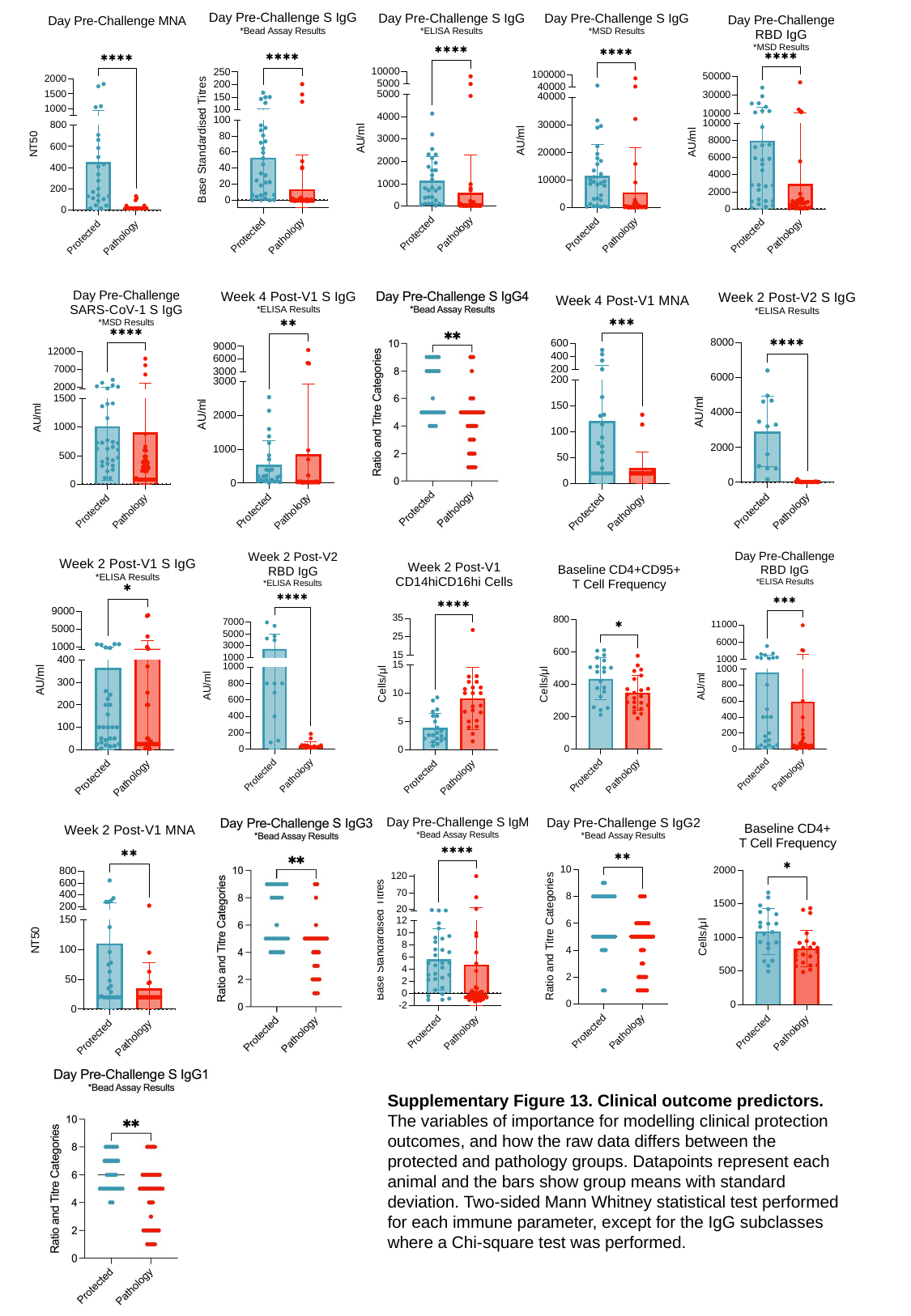

Supplementary Figure 13. Clinical outcome predictors. The variables of importance for modelling clinical protection outcomes, and how the raw data differs between the protected and pathology groups. Datapoints represent each animal and the bars show group means with standard deviation. Two-sided Mann Whitney statistical test performed for each immune parameter, except for the IgG subclasses where a Chi-square test was performed.

## Slide 15
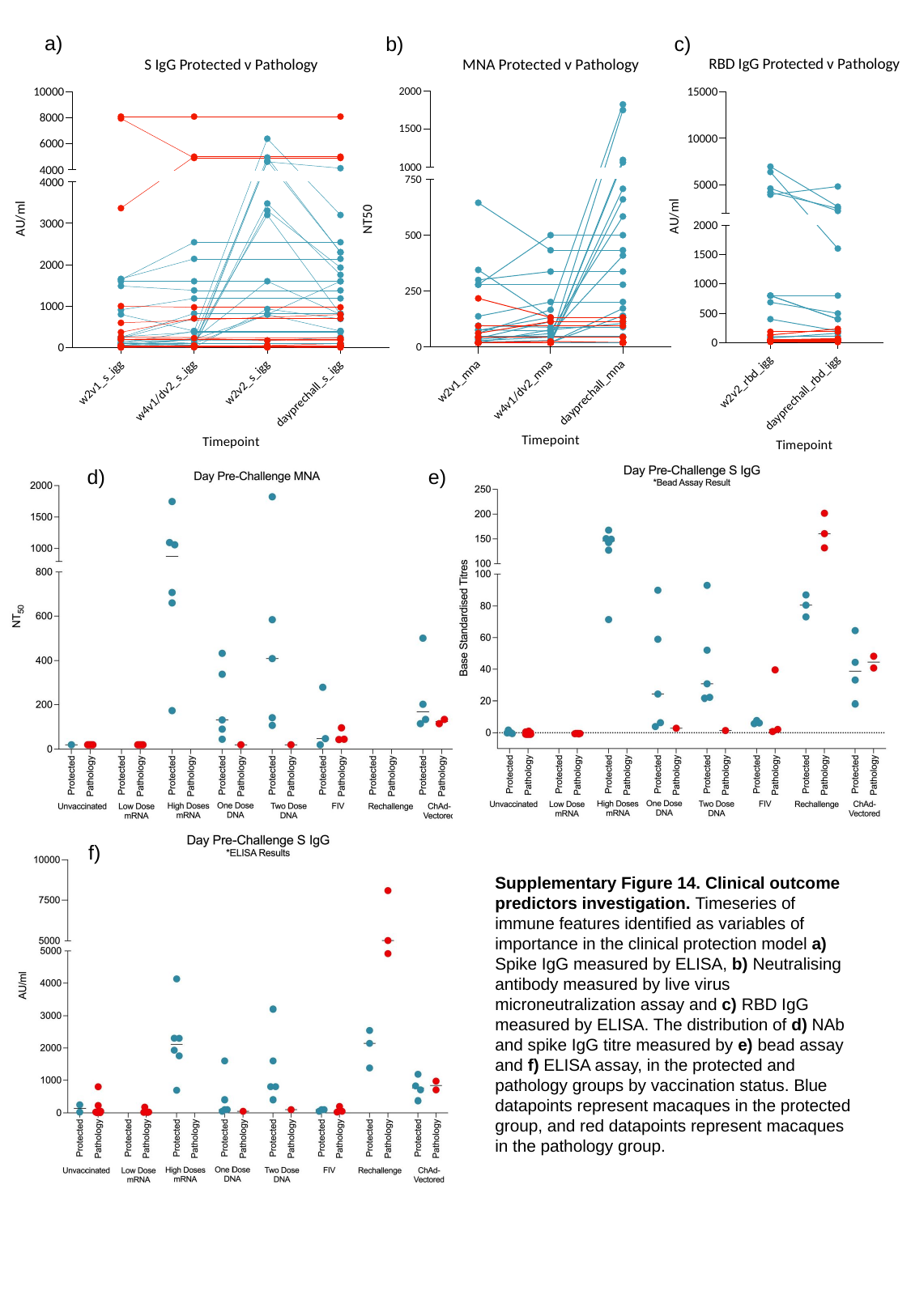

a)
c)
b)
d)
e)
f)
Supplementary Figure 14. Clinical outcome predictors investigation. Timeseries of immune features identified as variables of importance in the clinical protection model a) Spike IgG measured by ELISA, b) Neutralising antibody measured by live virus microneutralization assay and c) RBD IgG measured by ELISA. The distribution of d) NAb and spike IgG titre measured by e) bead assay and f) ELISA assay, in the protected and pathology groups by vaccination status. Blue datapoints represent macaques in the protected group, and red datapoints represent macaques in the pathology group.

## Slide 16
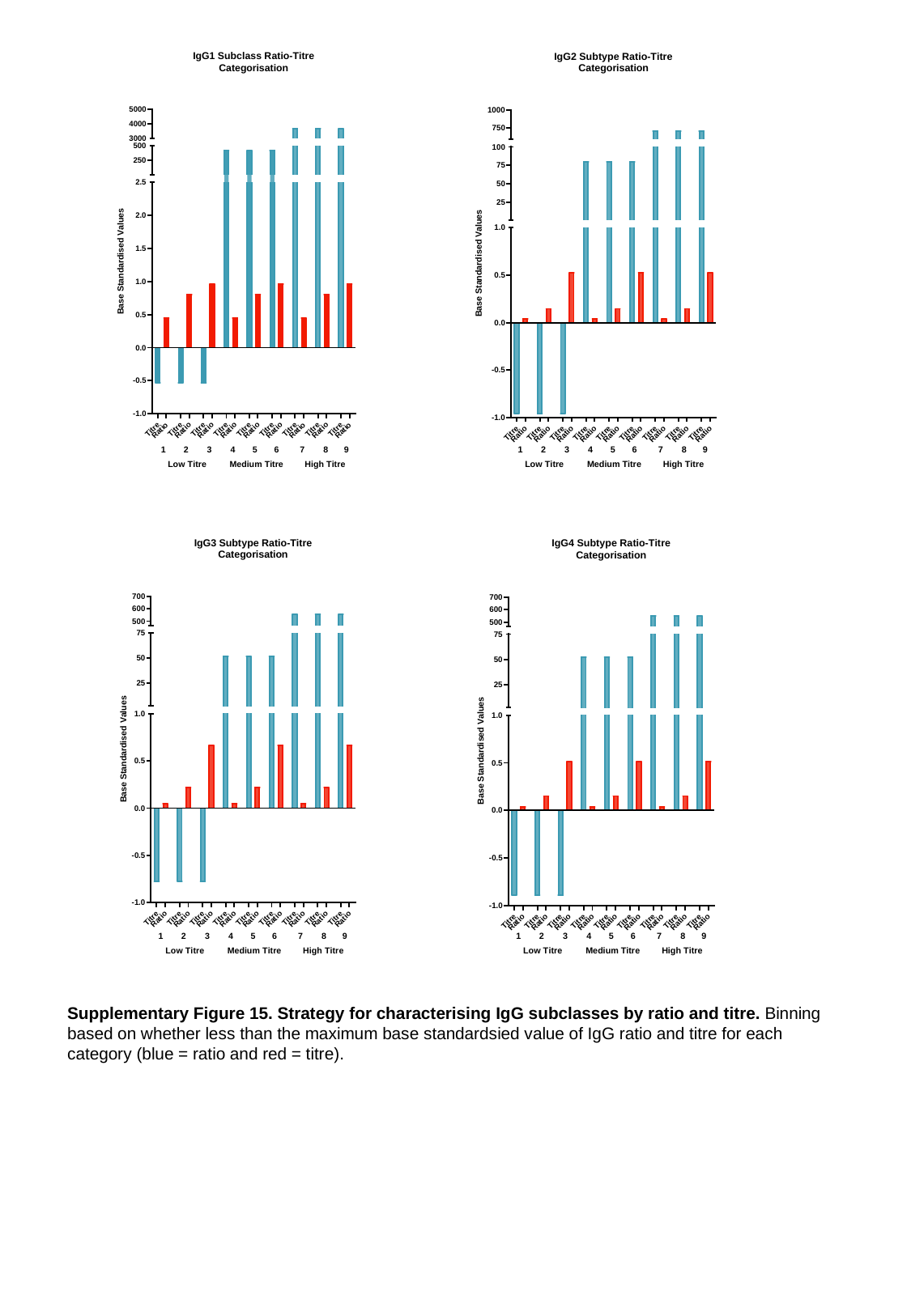

Supplementary Figure 15. Strategy for characterising IgG subclasses by ratio and titre. Binning based on whether less than the maximum base standardsied value of IgG ratio and titre for each category (blue = ratio and red = titre).

## Slide 17
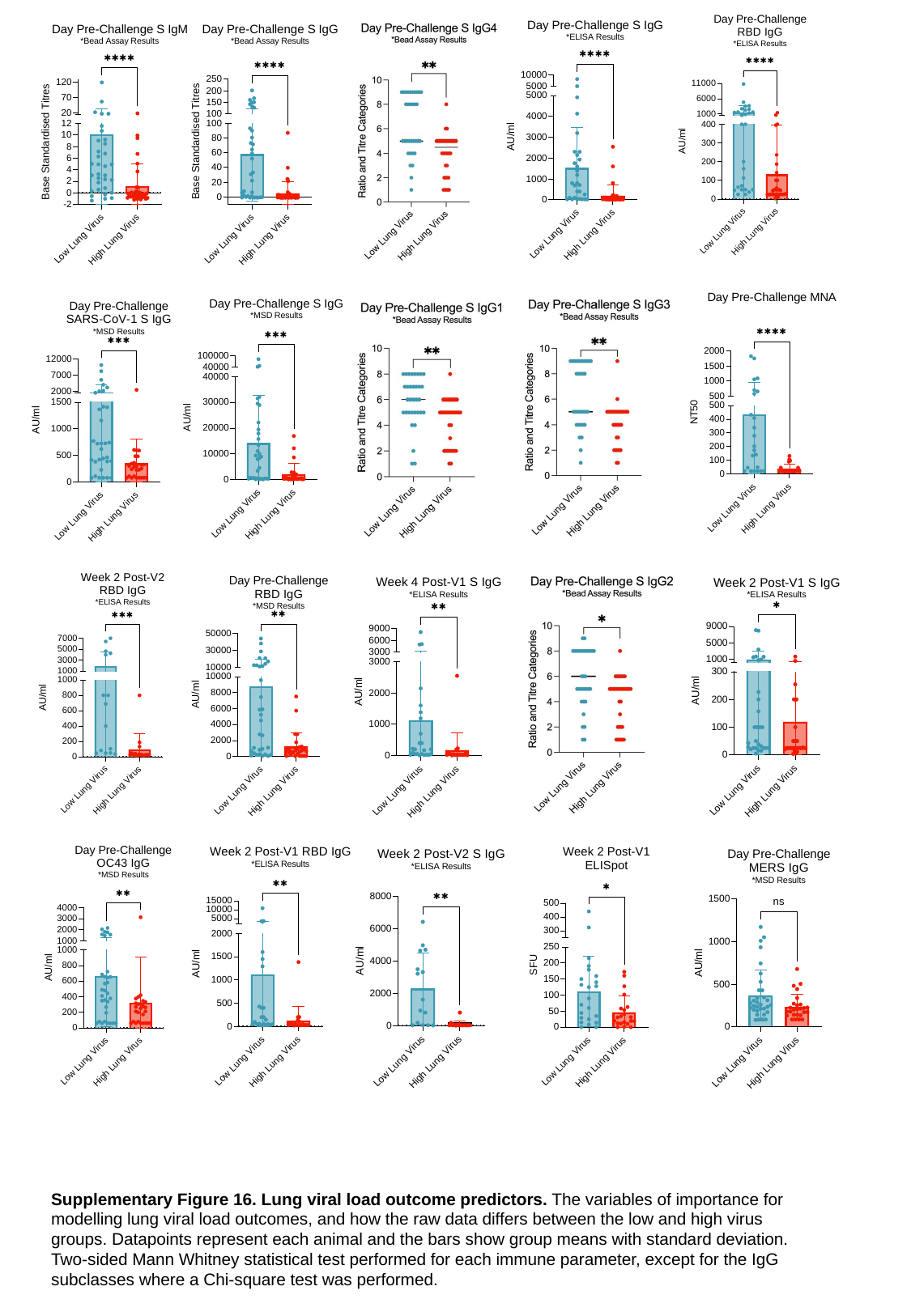

Supplementary Figure 16. Lung viral load outcome predictors. The variables of importance for modelling lung viral load outcomes, and how the raw data differs between the low and high virus groups. Datapoints represent each animal and the bars show group means with standard deviation. Two-sided Mann Whitney statistical test performed for each immune parameter, except for the IgG subclasses where a Chi-square test was performed.

## Slide 18
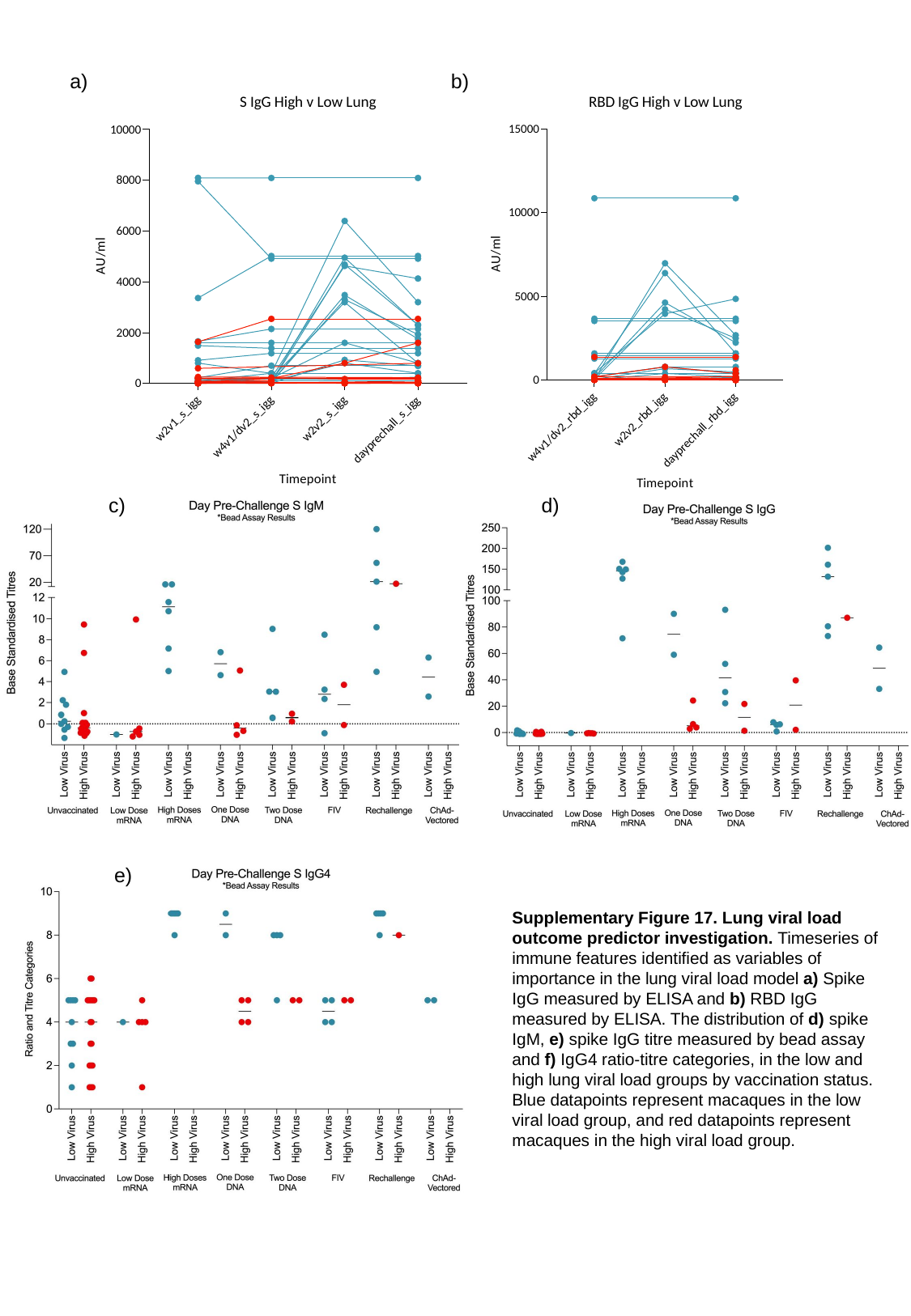

b)
a)
d)
c)
e)
Supplementary Figure 17. Lung viral load outcome predictor investigation. Timeseries of immune features identified as variables of importance in the lung viral load model a) Spike IgG measured by ELISA and b) RBD IgG measured by ELISA. The distribution of d) spike IgM, e) spike IgG titre measured by bead assay and f) IgG4 ratio-titre categories, in the low and high lung viral load groups by vaccination status. Blue datapoints represent macaques in the low viral load group, and red datapoints represent macaques in the high viral load group.

## Slide 19
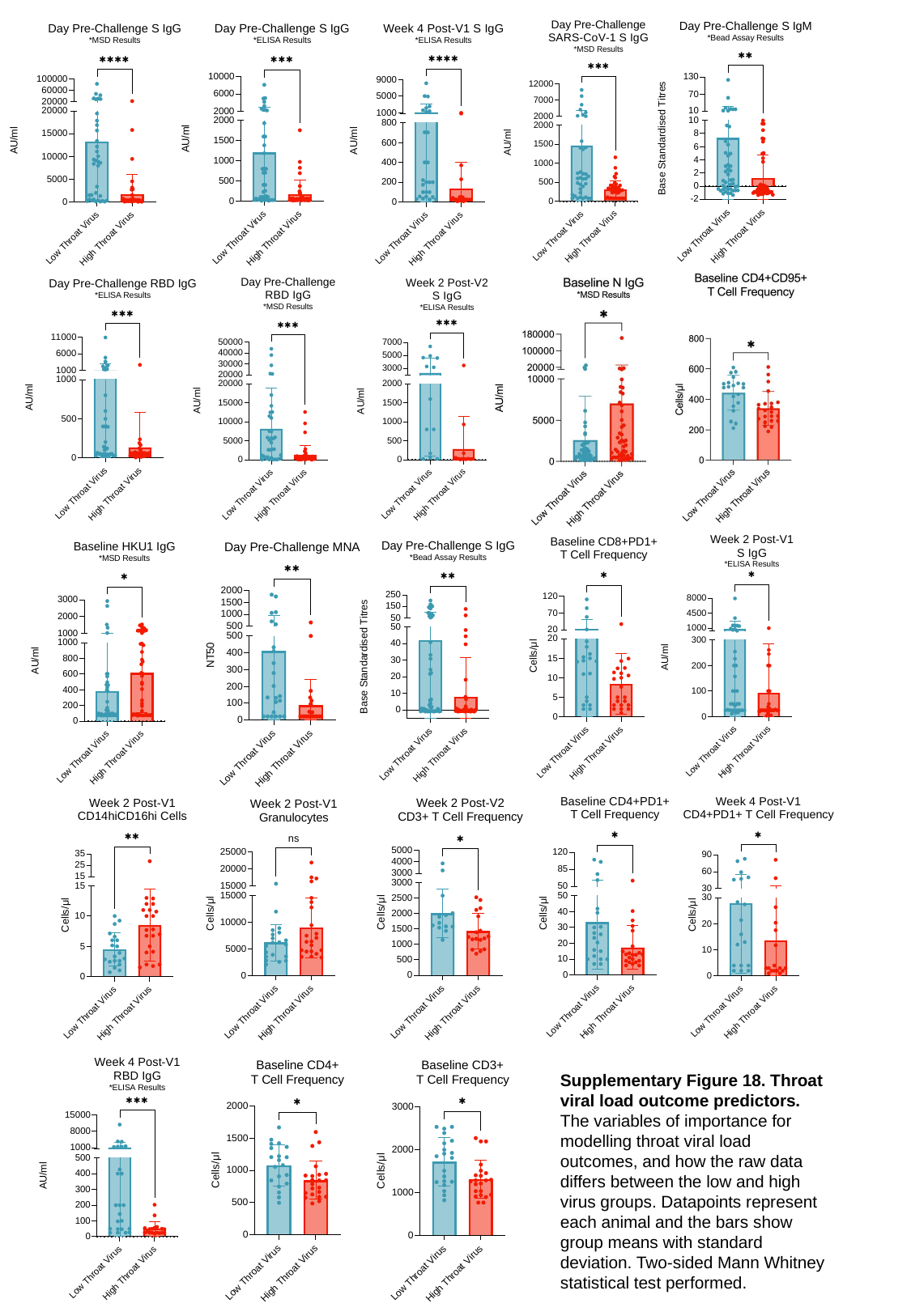

Supplementary Figure 18. Throat viral load outcome predictors. The variables of importance for modelling throat viral load outcomes, and how the raw data differs between the low and high virus groups. Datapoints represent each animal and the bars show group means with standard deviation. Two-sided Mann Whitney statistical test performed.

## Slide 20
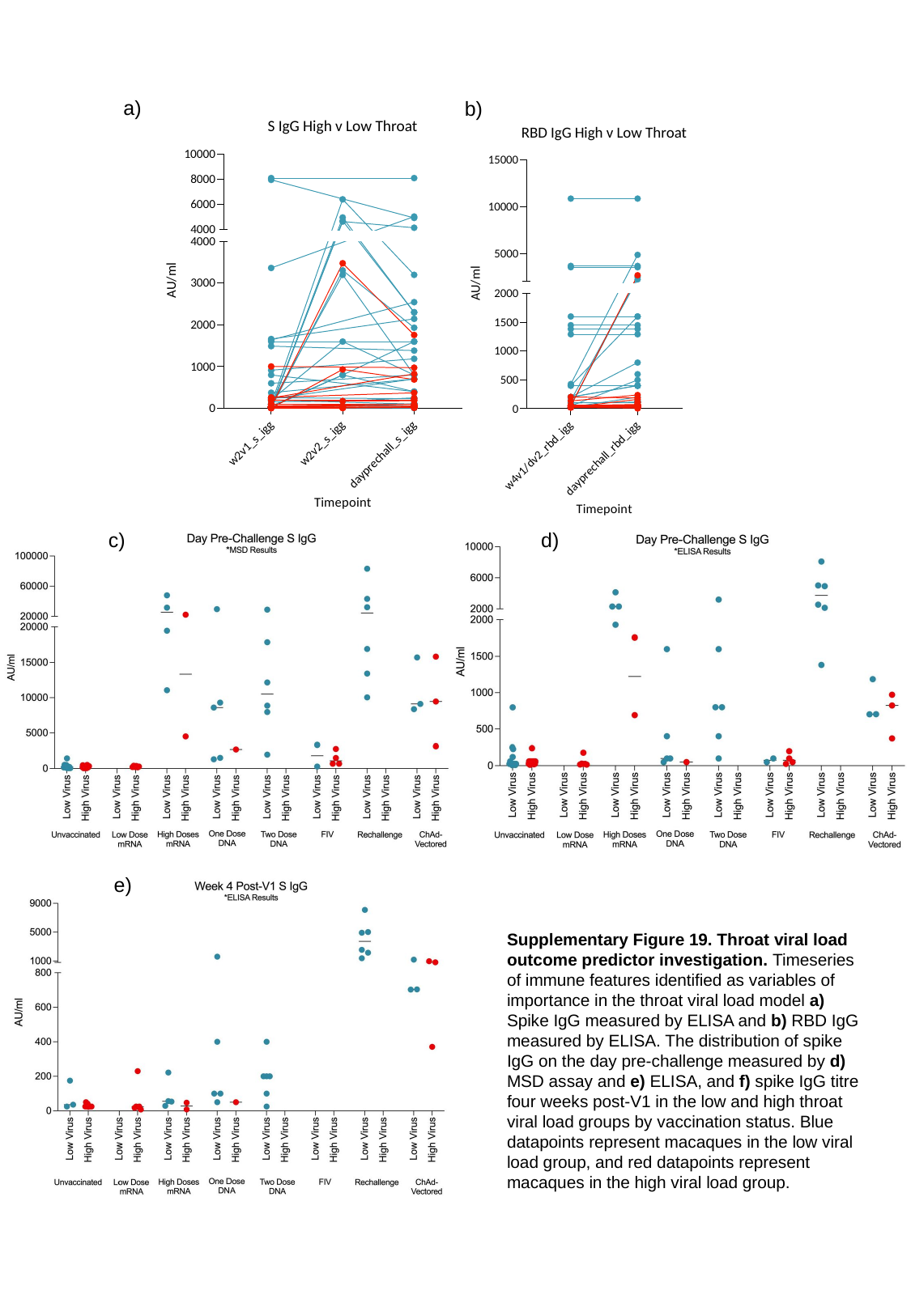

a)
b)
d)
c)
e)
Supplementary Figure 19. Throat viral load outcome predictor investigation. Timeseries of immune features identified as variables of importance in the throat viral load model a) Spike IgG measured by ELISA and b) RBD IgG measured by ELISA. The distribution of spike IgG on the day pre-challenge measured by d) MSD assay and e) ELISA, and f) spike IgG titre four weeks post-V1 in the low and high throat viral load groups by vaccination status. Blue datapoints represent macaques in the low viral load group, and red datapoints represent macaques in the high viral load group.

## Slide 21
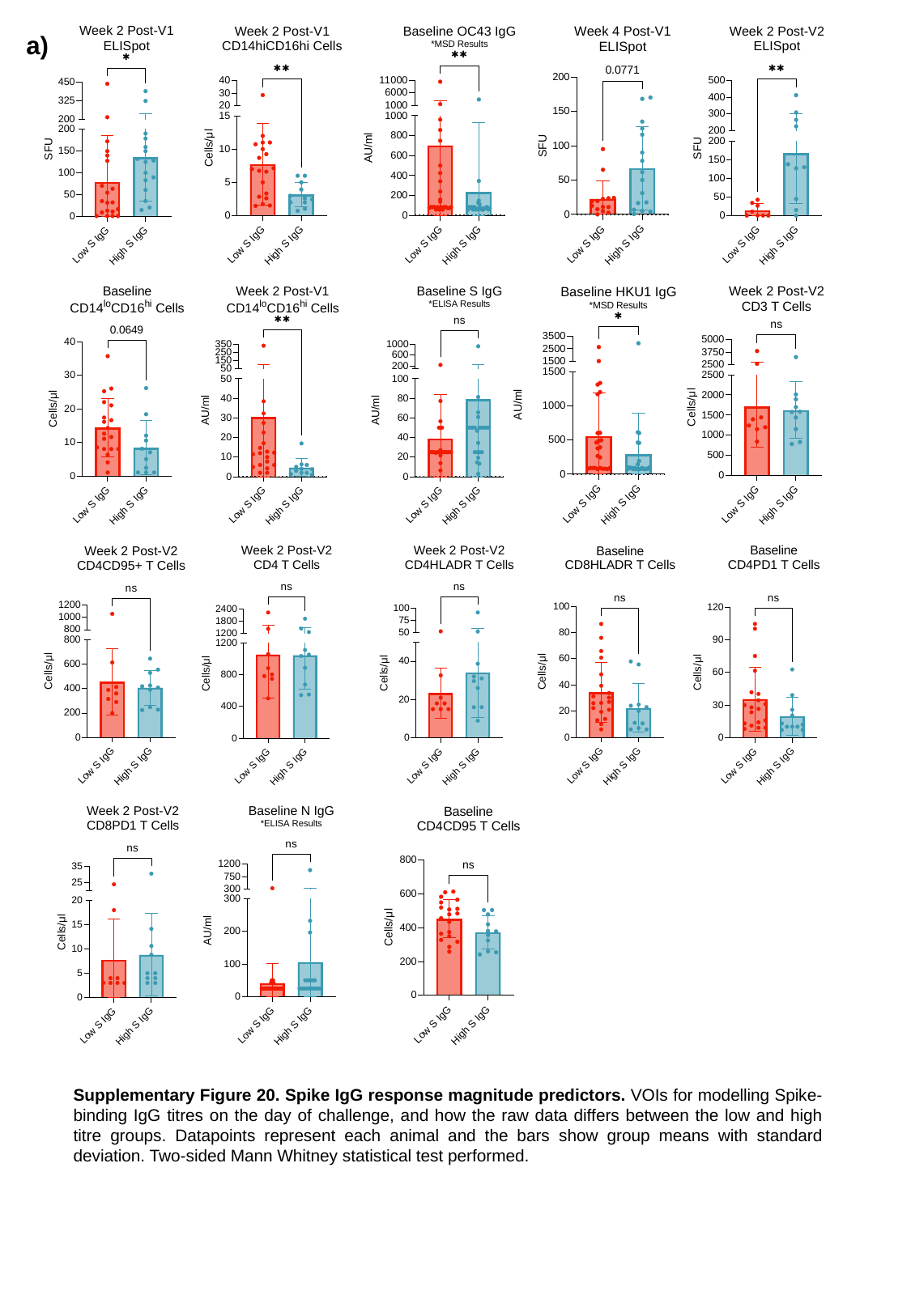

a)
Supplementary Figure 20. Spike IgG response magnitude predictors. VOIs for modelling Spike-binding IgG titres on the day of challenge, and how the raw data differs between the low and high titre groups. Datapoints represent each animal and the bars show group means with standard deviation. Two-sided Mann Whitney statistical test performed.

## Slide 22
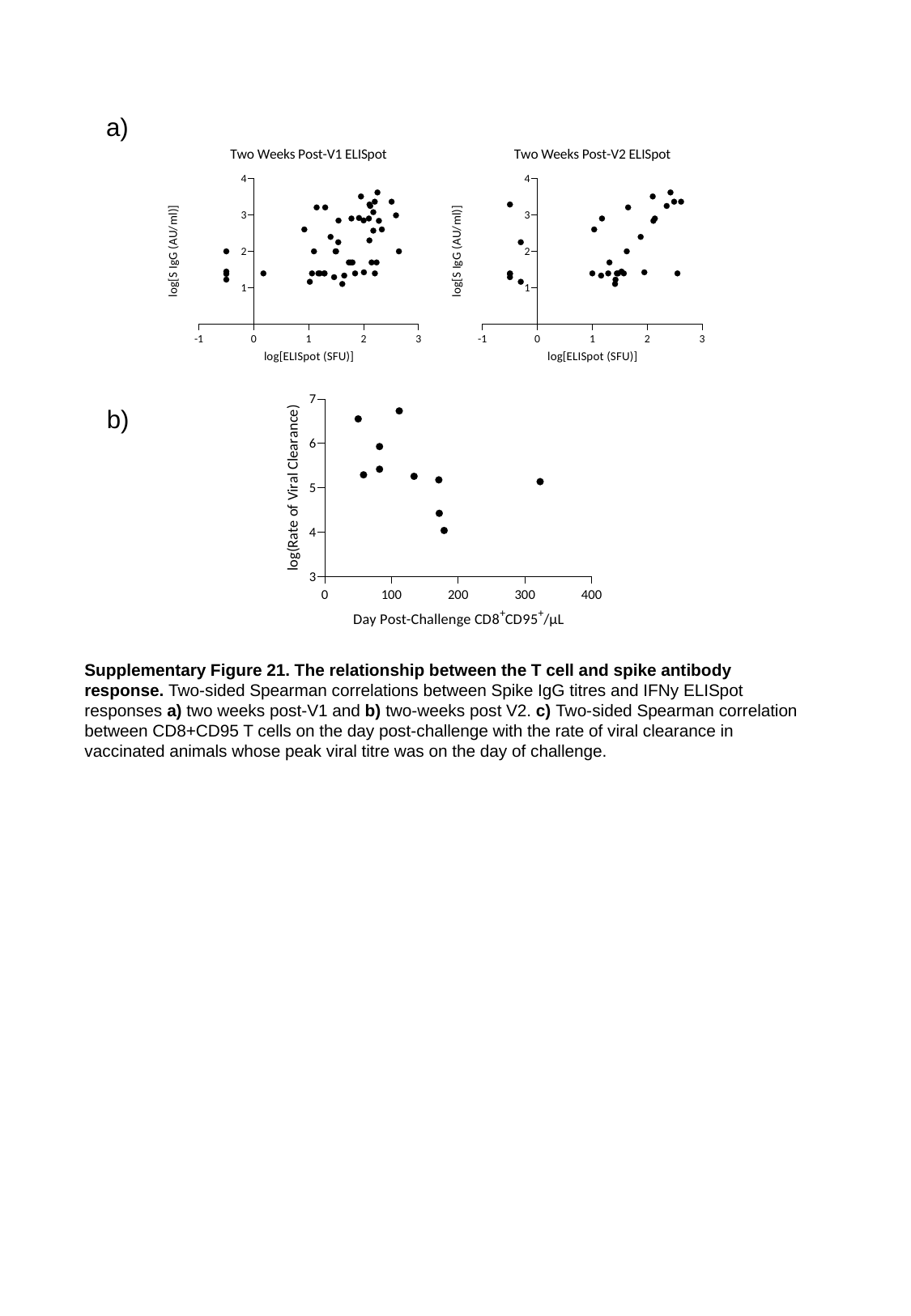

a)
b)
Supplementary Figure 21. The relationship between the T cell and spike antibody response. Two-sided Spearman correlations between Spike IgG titres and IFNy ELISpot responses a) two weeks post-V1 and b) two-weeks post V2. c) Two-sided Spearman correlation between CD8+CD95 T cells on the day post-challenge with the rate of viral clearance in vaccinated animals whose peak viral titre was on the day of challenge.

## Slide 23
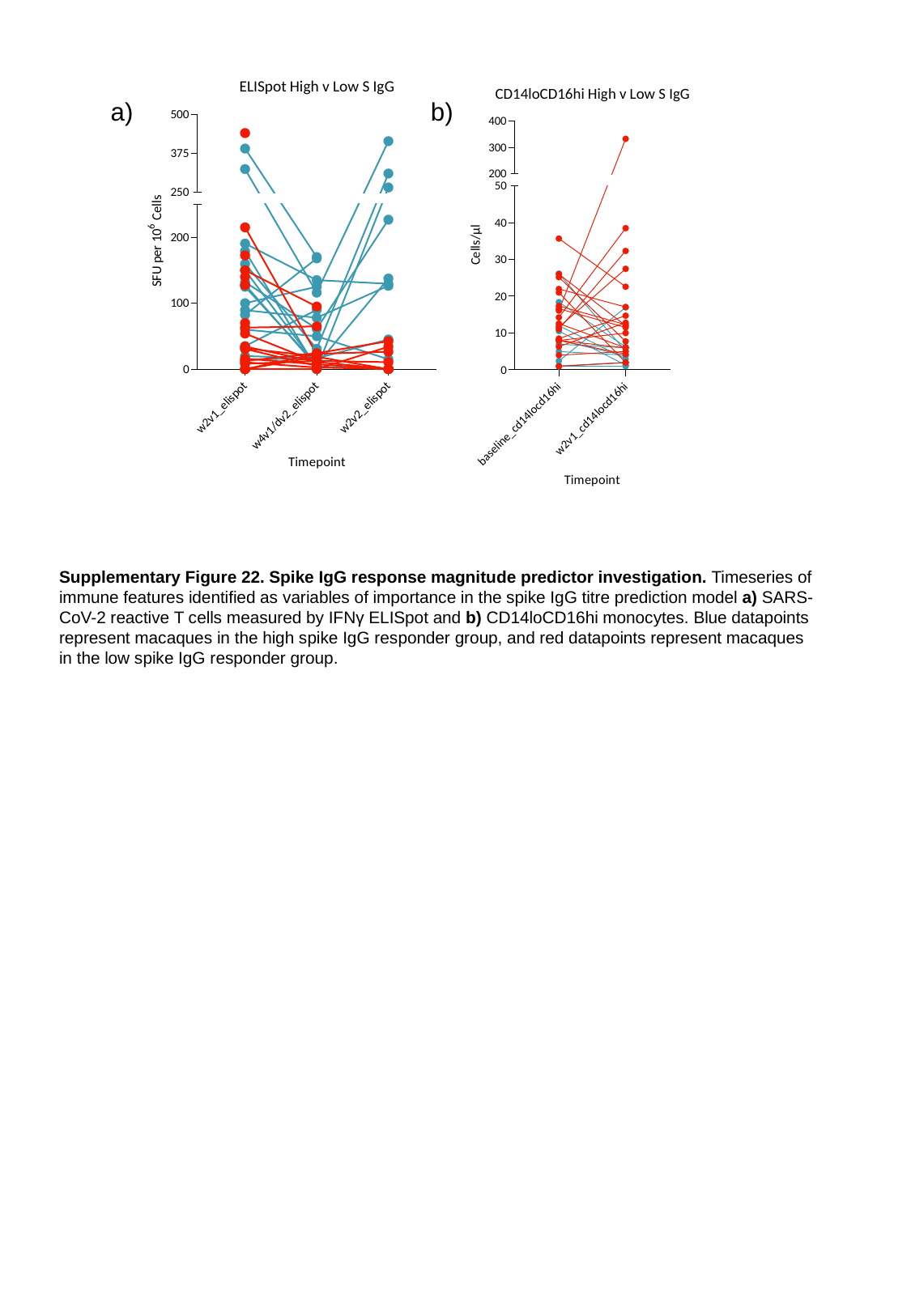

a)
b)
Supplementary Figure 22. Spike IgG response magnitude predictor investigation. Timeseries of immune features identified as variables of importance in the spike IgG titre prediction model a) SARS-CoV-2 reactive T cells measured by IFNγ ELISpot and b) CD14loCD16hi monocytes. Blue datapoints represent macaques in the high spike IgG responder group, and red datapoints represent macaques in the low spike IgG responder group.
